# Worldwide diversity and ecology of mangrove fungi: a systematic review of ITS metabarcoding studies and a quantitative, integrative analysis of raw sequence data

**DOI:** 10.1007/s11274-026-05065-y

**Published:** 2026-06-13

**Authors:** Isabela Maria Agustini da Silveira Bastos, Mariana Santos Cardoso, Marcele Laux, Rafael Rios Ribeiro, Glen Jasper Yupanqui García, Paloma Araujo Bahia, Paulo Miguel Vieira de Sousa, Bella Giselly Torres Alves, Diogo Henrique Costa de Rezende, Alexandre Soares Rosado, Jadson D.P. Bezerra, Melissa Fontes Landell, Vânia Maria Maciel Melo, Tallita Cruz Lopes Tavares, Aristóteles Góes-Neto

**Affiliations:** 1 Laboratory of Molecular and Computational Biology of Fungi (LBMCF), Department of Microbiology, Institute of Biological Sciences (ICB), Federal University of Minas Gerais (UFMG), Belo Horizonte, MG Brazil; 2 Instituto de Ciências do Mar (LABOMAR), Universidade Federal do Ceará (UFC), Fortaleza, CE Brazil; 3 Center for Genomics and Bioinformatics (CeGenBio), Federal University of Ceará, Fortaleza, CE Brazil; 4 Microbial Ecology and Biotechnology Laboratory (LEMBiotech), Department of Biology, Sciences Center, Federal University of Ceará, Fortaleza, CE Brazil; 5 King Abdullah University of Science and Technology (KAUST), Thuwal, Saudi Arabia; 6 Laboratório de Micologia (LabMicol), Instituto de Patologia Tropical e Saúde Pública (IPTSP), Universidade Federal de Goiás (UFG), Goiânia, GO Brazil; 7 Institute of Biological and Health Sciences, Federal University of Alagoas, Maceió, AL Brazil; 8 Department of Biochemistry, Institute of Chemistry, University of São Paulo, São Paulo, SP Brazil

**Keywords:** Intertidal forests, Amplicon metagenomics, Internal transcribed spacer, Fungi

## Abstract

Fungi are integral components of the mangrove microbiome, playing critical roles in decomposition, nutrient cycling, and symbiosis. Our study synthesizes the findings from a global systematic review of fungal ITS metabarcoding studies conducted in mangrove ecosystems. This review consolidates data from 23 original research articles (1,154 samples) and provides a comprehensive overview of the diversity, community structure, and ecological functions of fungi in these critical coastal habitats. The analyses revealed a consistent core fungal mycobiome in mangroves worldwide. This community is dominated by *Ascomycota*, with *Basidiomycota* as the second most abundant phylum. A consistent set of ten highly abundant genera underpins this core community, and fungal diversity and composition are strongly influenced by the specific substrate. Non-rhizospheric sediment harbors the highest diversity, while live plant organs host a more specialized and less diverse community, slightly dominated by potential plant pathogens. Rhizospheric sediment supports a unique assemblage rich in wood-decomposing fungi. The primary ecological role of fungi in mangroves is decomposition, which is essential for breaking down lignocellulosic litter, cycling nutrients, and storing carbon in sediments. A surprisingly high relative abundance of fungi classified as plant pathogens was identified on mangrove plant tissues, suggesting an underappreciated role of fungal diseases in these ecosystems. Metabarcoding provides a far broader view of fungal diversity than traditional collection and culturing methods. It has uncovered a vast number of uncultured taxa and has been particularly effective in revealing the significant, and likely underestimated, presence of macrofungi in mangrove soils. Our study also highlights that current short-read metabarcoding can severely underestimate certain fungal groups, particularly the endomycorrhizal *Glomeromycota*, due to technical limitations. Altogether, our synthesis provides a global baseline against which future mangrove mycobiome studies can be benchmarked.

**Graphical abstract:**

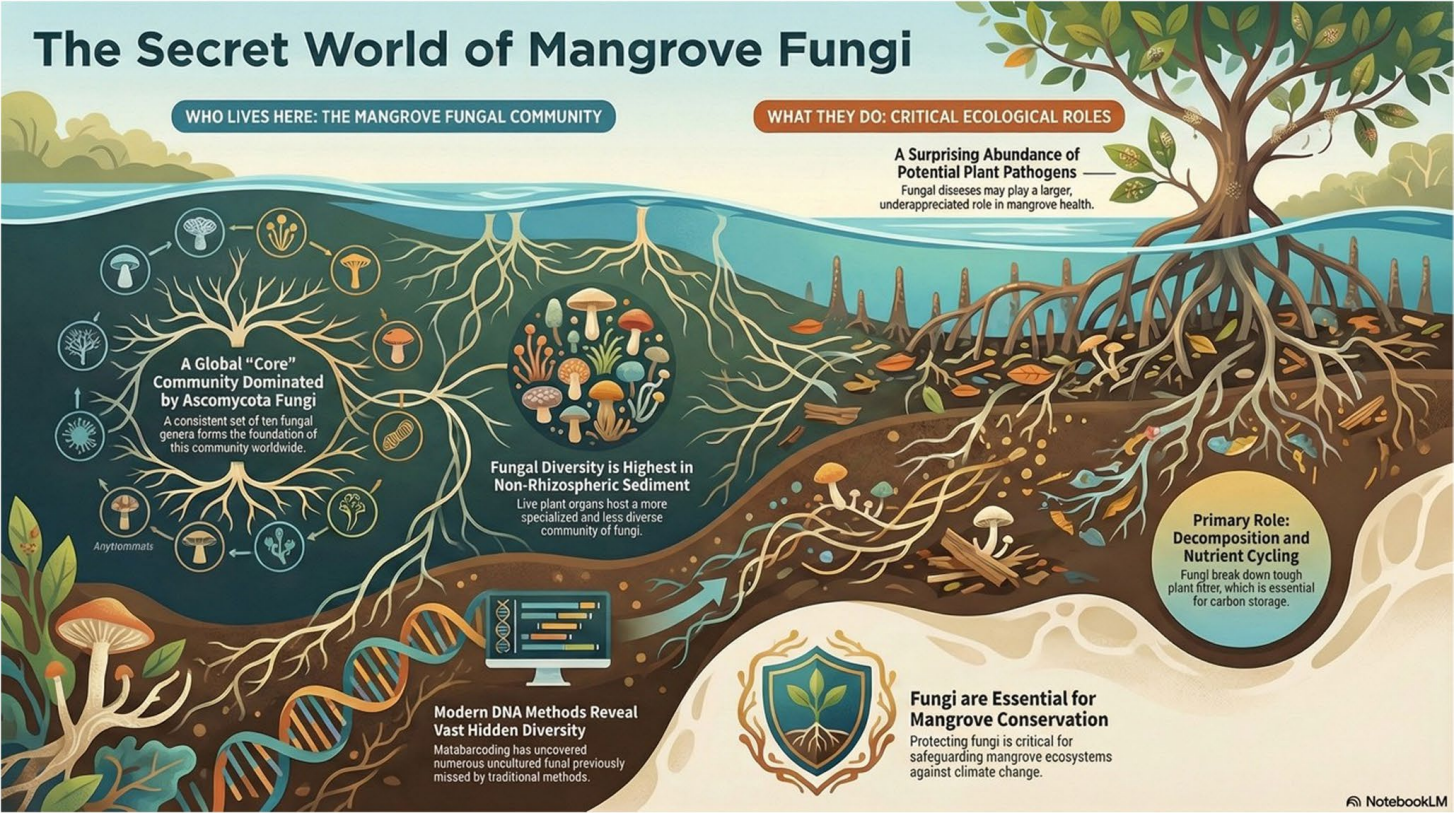

**Supplementary Information:**

The online version contains supplementary material available at 10.1007/s11274-026-05065-y.

## Introduction

Mangroves are coastal, intertidal forest ecosystems dominated by salt-tolerant trees and shrubs that thrive in brackish water and anoxic, muddy substrates and are subjected to daily tidal inundation (Lacerda et al. 2024; Friess et al. 2022; Kathiresan and Bingham 2001). Moreover, mangrove trees and shrubs exhibit hallmark adaptations to harsh environmental conditions, such as aerial, stilt, or pneumatophore roots, salt-exclusion and/or excretion mechanisms, and viviparous seedlings, which allow these plants to persist where few others can survive (Srikanth et al. 2016). In addition, their dense, sediment-trapping root mats stabilize shorelines and create structurally complex habitats along creeks and tidal channels (Christophe et al. 2021).

Geographically, mangrove forests occur along the tropical and subtropical shorelines of the Americas, Eurasia, and Africa, and Oceania, between 32° N and 38° S, spanning 124 countries and territories (Lacerda et al., 2024). Mangrove plant species are often arranged into zones governed by salinity gradients, inundation frequency, and wave exposure, ranging from seaward fringe forests to more sheltered riverine and basin stands (Lugo and Medina 2020). This ecological zonation reflects a tight coupling between plant physiology and local hydrogeomorphic conditions (Peters et al. 2020).

Mangroves constitute highly productive ecosystems in which leaf litter fuels the detrital food chain that supports diverse invertebrate communities (Lee, 2008). At the same time, their intricate root systems provide refuge and foraging habitat for fish, birds, and other animals. Biogeochemically, mangroves are notable “blue carbon” ecosystems, as they store substantial carbon stocks in above- and belowground biomass and, particularly, in deep organic soils, where residence times can be considerably long (Meng et al. 2021). By attenuating wave energy and trapping fine sediments, mangroves also accrete land and improve nearshore water quality by retaining nutrients and pollutants (Sarker et al. 2021).

All those aforementioned ecological functions translate into substantial ecosystem services. Mangroves attenuate wave energy and storm surges, reduce erosion and flood damage, and serve as cost-effective natural infrastructure for climate adaptation (Ayassamy 2025). As a critical nursery habitat, they sustain artisanal and commercial fisheries that support human communities worldwide (Huxham et al. 2017). Besides, their high carbon density and sequestration rates make them priority candidates for nature-based climate solutions that deliver both mitigation and resilience cobenefits (Hilmi et al. 2025).

Despite a slowing in global net loss rates in recent years, mangroves remain threatened. Direct conversion to shrimp aquaculture, urban and port development, and agriculture has substantially fragmented many coastlines (Ferreira et al. 2022). Hydrological alteration, primarily through dams, freshwater diversions, and/or channel modifications, disrupts salinity and sediment supply, whereas degraded water quality imposes additional stress (Mojid et al. 2025). Hence, climate change compounds these pressures via sea-level rise, marine heatwaves, and more intense storms (Zhou et al. 2025).

Fungi are integral components of the mangrove microbiome, playing critical roles in decomposition, nutrient cycling, and symbiosis (Britto Martins de Oliveira et al., 2025). The taxonomic composition of this community is diverse, with studies identifying representatives from most of the known fungal phyla (Lee et al. 2019). Furthermore, recent advancements in cultivation and sequencing technologies have expanded our understanding of this diversity (Zhang et al. 2024b). For example, a study employing high-throughput iChip cultivation isolated more than 700 fungal strains from mangrove sediments, 70% of which were cultivable only by this advanced method (Li et al. 2023). Studies employing novel approaches to retrieve most of the fungi occurring in mangroves highlight the existence of a vast, largely unknown fungal community composed of unculturable lineages that were historically overlooked.

The study of fungi in mangrove ecosystems has a long, but historically narrow, intellectual lineage. The earliest reports date from the first half of the past century, with Stevens (1920) describing mangrove fungi from Puerto Rico and Cribb and Cribb (1955) from Australia. Subsequent decades saw a gradual expansion driven primarily by morphology-based marine mycology (Kohlmeyer 1969; Hyde et al. 1998), culminating in the foundational global compendium of marine and mangrove ascomycetes assembled by Schmit and Shearer (2004) and, more recently, in the comprehensive global checklist of Devadatha et al. (2021), which represents the most exhaustive synthesis of mangrove fungal occurrence and geographical distribution available to date. From a taxonomic standpoint, this body of work has consistently identified mangroves as Ascomycota-dominated systems, with Basidiomycota as the second most abundant phylum and an increasing recognition of basal lineages such as Chytridiomycota and Rozellomycota in molecular surveys (Lee et al. 2019; Zhang et al. 2024b). Functionally, mangrove fungi are currently understood to occupy four broad ecological roles: (i) saprotrophy on lignocellulosic plant necromass, the dominant lifestyle in sediments and a key driver of carbon and nutrient cycling; (ii) endophytism in living plant tissues, which contributes to host stress tolerance and to the production of bioactive secondary metabolites; (iii) plant pathogenicity, increasingly suspected to underpin still-undescribed mangrove diseases; and (iv) mycorrhizal symbiosis, encompassing both ectomycorrhizal and endomycorrhizal associations relevant to nutrient acquisition under the saline, hypoxic and oligotrophic conditions characteristic of mangrove sediments (Britto Martins de Oliveira et al. 2025; Kundu et al. 2025). Despite this conceptual maturity, the overwhelming majority of the historical record relies on direct field collection and on culture-dependent isolation, which together capture only a small fraction of the actual fungal diversity present in any given substrate (Li et al. 2023).

Although Devadatha et al. (2021) provided an excellent global compendium of mangrove fungi based on direct field collection and culture-dependent isolation, no equivalent global synthesis exists for ITS-amplicon-based metabarcoding studies. Furthermore, the few existing meta-syntheses do not re-analyse raw reads under a unified bioinformatic pipeline, making cross-study comparisons unreliable. By filling this gap with a globally inclusive, PRISMA-guided synthesis combined with re-analysis of public raw reads under a single bioinformatic pipeline, our study provides the first standardised baseline of fungal diversity, structure and functional traits in mangrove ecosystems worldwide, a baseline that is essential for conservation planning, climate-change-impact monitoring, and the rational design of fungal-consortium-based restoration strategies.

Hence, the main objective of our study was to conduct a global systematic review of fungal metabarcoding (ITS) studies in mangroves and; therefore, to produce a scientific synthesis of current knowledge on fungal diversity in these highly endangered and crucial ecosystems in the context of climate change.

By filling this gap with a globally inclusive, PRISMA-guided synthesis combined with re-analysis of public raw reads under a single bioinformatic pipeline, our study provides the first standardised baseline of fungal diversity, structure and functional traits in mangrove ecosystems worldwide, a baseline that is essential for conservation planning, climate-change-impact monitoring and the rational design of fungal-consortium-based restoration strategies.

## Materials and methods

The systematic review process was developed in accordance with the Preferred Reporting Items for Systematic Reviews and Meta-Analyses (PRISMA) guidelines (Page et al. 2021). The study comprised the following main stages: (1) identification of records, which involved a comprehensive search across three major scientific databases to retrieve potentially relevant studies; (2) screening of records, including the automatic removal of duplicate records, the exclusion of documents without DOI, documents unavailable for reading and that did not represent original research (such as review articles, systematic reviews, books, and book chapters); (3) assessment of studies based on predefined eligibility and exclusion criteria, in which the titles and abstracts were initially screened using the Rayyan.ai platform, followed by full-text reading and careful evaluation to determine their eligibility; and (4) synthesis and analysis of data. The PRISMA checklist (Page et al. 2021) was used to guide and describe the items for each step (**Supplementary Material **1**).**

### Identification of records

For this review, records were extracted from publications indexed in the PubMed, Scopus, and Web of Science databases by searching for combined terms (keywords) using the Boolean operators “OR” and “AND” (**Supplementary Material **2**).** For the Scopus and PubMed databases, a local script developed by co-author Glen García (GG) (García et al. 2025) on a Python 3.10.8 system, using the MySQL database manager (https://github.com/LBMCF/scientific_database), was used to retrieve the records included in this review. For the Web of Science database, records were manually extracted from publications indexed using the keywords provided in **Supplementary Material **2. The most recent database access was on 31 June 2025 (**Supplementary Material **3). The studies included all types of documents, in all languages, and without publication date restrictions.

### Screening of records through both automated tools and manual review

In the second phase of the document selection process, the data for the records identified in the Scopus and PubMed databases were exported as CSV files and then converted to .xlsx using the *format_input.py* script (https://github.com/lbmcf/format-input). The *script* identified records without DOIs and removed those with identical titles or DOIs **(Supplementary Material **3**)**. Duplicate records in the .xlsx files containing unified records were removed using the script remove_duplicates.py (**Supplementary Material **3**).**

### Selection, screening, exclusion, and inclusion criteria of studies

Based on the study screening, data from records identified in the Scopus and PubMed databases (**Supplementary Material **3) were downloaded as CSV files from Excel and exported to the website https://new.rayyan.ai/. The authors manually reviewed the titles, abstracts, and keywords. After reviewing the documents, the preselected studies were thoroughly re-evaluated using the eligibility and exclusion criteria listed in Table 1. The evaluation of the exclusion and inclusion criteria were (1) collection point; (2) sample type; (3) organism; (4) sequencing method.

**Table 1:** Exclusion and inclusion criteria of selection studies in the systematic review

Inclusion criteria
1: Collection point: Mangrove or microcosms of mangrove
2: Sample type: non rhizosphere sediment or soil collected in an area with vegetation; sediment or soil collected in a non-vegetated area; rhizospheric soil; sediments kept in microcosms without vegetation and parts of plant (leaves, fruits, pneumatophores- aerial root, trunks, roots, stem). *Samples contained in plant areas or taken from parts of the plant selected from native and associated mangrove species
3: Organism: Fungi
4: Sequencing method and region of the microorganism’s gene: Amplicon sequencing and of the ITS region
Exclusion criteria
1: Sample: Obtained from cultures
2: Studies that did not present molecular methods or microbiological analysis
3: Scientific studies: book chapters, book, review articles, and posters 4: Studies without DOI

### Data extraction of selected studies

The following data were extracted from selected documents by authors IB, Paloma Bahia (PB), and TT (**Supplementary Material **4**).** Chosen data: (1) Characteristics of the Studies: ID of article, year of publication, journal published (Table 2); (2) Sampling: mangrove geographic regions, countries, sample type, number of sequenced samples (Fig. 2; Table 2, and **Supplementary Material **5); (3) Molecular Data and (4) Taxonomic Composition.

**Table 2:** Studies selected and their characteristics by journal published and impact factor, mangrove geographic regions, countries, sample type, and number of samples of sampling

Reference	Journal published	Geographic Region	Country	Sample Code	Sample Type	Number of samples sequenced	ID Article
Yu et al. (2023)	Chemosphere	EAs	China	SMP_A	Sediments kept in microcosms without vegetation	10	3
				SMP_B	Sediments were kept in microcosms without vegetation and contaminated with hydrocarbons.	12	
Zhou et al. (2021)	Frontiers in Microbiology	EAs	China	SMP_C	Non-rhizospheric sediment or soil collected in areas with vegetation	12	64
				SMP_D	Rhizospheric Soil	12	
				SMP_E	parts of plants (root episphere and endosphere)	24	
Zhang et al. (2021)	Applied and Environmental Microbiology	EAs	China	SMP_C	Non-rhizospheric sediment or soil collected in areas with vegetation	107	73
				SMP_F	Sediment or soil collected in a non-vegetated area	3	
Wang et al. (2021)	Journal of Hazardous Materials	EAs	China	SMP_C	Non-rhizospheric sediment or soil collected in an area with vegetation	3	77
				SMP_D	Rhizospheric Soil	3	
				SMP_E	parts of plants (roots, episphere, and endosphere)	6	
Reference	**Journal published**	**Geographic Region**	**Country**	**Sample Code**	**Sample Type**	**Number of samples sequenced**	**ID Article**
Zhuang et al. (2020)	Biofilms and Microbiomes	EAs	China	SMP_C	Non-rhizospheric sediment or soil collected in an area with vegetation	6	91
				SMP_D	Rhizospheric Soil	6	
				SMP_E	parts of plants (root episphere and endosphere)	12	
Lee et al. (2020)	IMA Fungus	SEAs	Malaysia	SMP_C	Non-rhizospheric sediment or soil collected in an area with vegetation	41	97
				SMP_E	parts of plants (leaves, fruits, and pneumatophores - aerial roots)	140	
			Singapore	SMP_C	Non-rhizospheric sediment or soil collected in an area with vegetation	40	
				SMP_E	parts of plants (leaves, fruits, and pneumatophores - aerial roots)	130	
Yao et al. (2019)	Microbiome	EAs	China	SMP_E	parts of plants (leaves)	48	135
Chi et al. (2019)	PeerJ	EAs	Taiwan	SMP_E	parts of plants (leaves)	17	141
Cheung et al. (2018)	Scientific Reports	EAs	China	SMP_C	Non-rhizospheric sediment or soil collected in an area with vegetation	18	145
Arfi et al. (2012)	FEMS Microbiology Ecology	Mel	New Caledonia	SMP_E	parts of plants (leaves, trunks, and branches)	12	236
Reference	**Journal published**	**Geographic Region**	**Country**	**Sample Code**	**Sample Type**	**Number of samples sequenced**	**ID Article**
Lee et al. (2019)	Frontiers in Microbiology	SEAs	Malaysia	SMP_C	Non-rhizospheric sediment or soil collected in an area with vegetation	30	264
				SMP_E	parts of plants (leaves, fruits, and pneumatophores - aerial roots)	90	
			Singapore	SMP_C	Non-rhizosphere sediment or soil collected in area with vegetation	40	
				SMP_E	parts of plants (leaves, fruits, and pneumatophores - aerial roots)	119	
Haldar and Nazareth (2019)	3 Biotech	SAs	India	SMP_C	Non-rhizosphere sediment or soil collected in area with vegetation	2	266
Myovela et al. (2025)	Biologia	EAf	Tanzania	SMP_C	Non-rhizosphere sediment or soil collected in area with vegetation	2	333
				SMP_E	parts of plants (leaves, roots, and stems)	8	
Yang et al. (2024)	Microbiome	EAs	China	SMP_E	parts of plants (leaves)	36	336
Mahanty et al. (2024)	Marine Pollution Bulletin	SAs	India	SMP_C	Non-rhizospheric sediment or soil collected in an area with vegetation	2	338
				SMP_F	Sediment or soil collected in non-vegetated area	2	
Reference	**Journal published**	**Geographic Region**	**Country**	**Sample Code**	**Sample Type**	**Number of samples sequenced**	**ID Article**
Shah et al. (2024)	Ecological Genetics and Genomics	SAs	India	SMP_C	Non-rhizospheric sediment or soil collected in an area with vegetation	1	368
				SMP_E	parts of plants (leaves, roots, and stems)	3	
Muwawa et al. (2024)	Plos One	EAf	Kenya	SMP_D	Rhizosphere soil	64	379
Lin et al. (2024)	Environmental Pollution	EAs	China	SMP_G	Sediments were maintained in microcosms without vegetation and were supplemented with micro-sized polypropylene.	3	380
Li et al. (2024)	Acta Microbiologica Sinica	EAs	China	SMP_D	Rhizosphere soil	9	394
Zhang et al. (2024a)	Frontiers in Earth Science	EAs	China	SMP_C	Non-rhizospheric sediment or soil collected in an area with vegetation	54	397
Costa et al. (2020)	Scientific Reports	SAm	Brazil	SMP_C	Non-rhizospheric sediment or soil collected in an area with vegetation	18	443
Vanegas et al. (2019)	Fungal Ecology	SAm	Colombia	SMP_D	Rhizosphere soil	9	448
Yu et al. (2022)	Journal of Hazardous Materials	EAs	China	SMP_A	Sediments were kept in microcosms without vegetation.	10 (same article ID 3)	480
				SMP_B	Sediments were kept in microcosms without vegetation and contaminated with hydrocarbons.	12 (same article ID 3)	

### Data analyses

#### Sampling mangrove geographic regions and sample types

Data analysis was carried out for each sample type (SMP) separately (**SMP**_**A**: Sediments kept in microcosms without vegetation; **SMP**_**B**: Sediments kept in microcosms without vegetation and contaminated with hydrocarbons; **SMP**_**C**: Non-rhizospheric sediment or soil collected in area with vegetation; **SMP**_**D**: Rhizospheric Soil; **SMP**_**E**: parts of the native and associated mangrove plant species (leaves/fruits/ pneumatophores- aerial roots/trunks/roots/stems); **SMP**_**F**: sediment or soil collected in non-vegetated area and **SMP**_**G**: Sediments kept in microcosms without vegetation added with micro-sized Polypropylene**).** Each set of sample types was evaluated for each mangrove geographic region following the classification of Global Mangrove Watch (GMW) (Bunting et al. 2022) and were indicated by: EAf-East Africa; MAf-Middle Africa; SAf-Southern Africa; WAf-Western Africa; Car-Caribbean; CAm-Central America; SAm- South America; NAm-Northern America; EAs-Eastern Asia; SEAs-Southeastern Asia; SAs-Southern Asia; WAs-Western Asia; ANZ-Australia & New Zealand; Mel-Melanesia; Mic-Micronesia and Pol- Polynesia.

#### Molecular methods and taxonomic assessment

Data on molecular materials and methods from the selected articles were synthesized to identify patterns in sample processing. This included sample preprocessing and preservation methods (Table 3), protocols for DNA extraction and amplification, and the specific sequencing platforms used (Table 4). Taxonomic composition analysis and determination of absolute and relative abundance of microorganisms were performed using ITS rRNA amplicon sequencing data. Notably, these downstream analyses—including taxonomic abundance, alpha diversity, and beta diversity—were explicitly performed at the genus level. Furthermore, these analyses were restricted exclusively to datasets generated using the Illumina platform to ensure methodological consistency and data comparability across studies. Regarding the sample types, **SMP_F** (sediment/soil from non-vegetated areas) and **SMP_G** (microcosm sediments with polypropylene) were not evaluated for genus-level abundance or diversity indices. This exclusion was necessary because the raw sequencing data for these samples were either unavailable or not publicly available in databases. However, these samples were also fully included in the other analyses in this study, ranging from study characteristics to the molecular materials and methods used.

**Table 3:** Methods of preprocessing and preservation from sample types for DNA extraction and sequencing included in the selected studies in this systematic review

Sample Type	Samples Characteristics	Number of samples totals	Sample storage methods for DNA extraction at the collection site	DNA Preprocessing Methods	Sample Storage Methods for DNA extraction in the laboratory/ DNA Preservation Agent	ID Article	Reference
SMP_A	Sediments kept in microcosms without vegetation	10	Not declared	**(i)** Incubation of sediment samples in uncontaminated microcosms at room temperature (25 °C) for up to 20 months, after collection and homogenization of samples.	Not declared	3 and 480 (complementary articles)	Yu et al. (2023) and Yu et al. (2022)
SMP_B	Sediments were kept in microcosms without vegetation and contaminated with hydrocarbons.	12	Not declared	**(i)** Incubation of sediment samples in hydrocarbon-contaminated microcosms (HBCDs) at room temperature (25 °C) for up to 20 months, after collection and homogenization of the samples.	Not declared	3 and 480 (complementary articles)	Yu et al. (2023) and Yu et al. (2022)
SMP_C	Non-rhizospheric sediment or soil collected in areas with vegetation	81	Not declared	(**ii**) Homogenization (Omni Bead Ruptor 24 (Omni International, Kennesaw, GA, United States at 8 m/s for 2 min)	Not declared	97	Lee et al. (2020)
SMP_C	Non-rhizospheric sediment or soil collected in an area with vegetation	70	Not declared	(**ii**) Homogenization (Omni Bead Ruptor 24 (Omni International, Kennesaw, GA, United States at 8 m/s for 2 min)	Not declared	264	Lee et al. (2019)
SMP_C	Non-rhizospheric sediment or soil collected in an area with vegetation	18	Ice box	(**iii**) Sieved through a 2-mm	Stored at -80 °C	145	Cheung et al. (2018)
SMP_C	Non-rhizospheric sediment or soil collected in areas with vegetation	12	Not declared	(**iv**) Shaken off the root	Stored at -80 °C	64	Zhou et al. (2021)
SMP_C	Non-rhizospheric sediment or soil collected in areas with vegetation	3	Portable cooler at 4 °C	(**iv**) Shaken off the root	Stored at -80 °C	77	Wang et al. (2021)
SMP_C	Non-rhizospheric sediment or soil collected in areas with vegetation	6	Not declared	(**iv**) Shaken off the root	Stored at -80 °C	91	Zhuang et al. (2020)
SMP_C	Non-rhizospheric sediment or soil collected in areas with vegetation	107	Ice box	Not declared	Stored at -40 °C	73	Zhang et al. (2021)
SMP_C	Non-rhizospheric sediment or soil collected in an area with vegetation	2	Not declared	Not declared	Stored at − 20 °C	266	Haldar and Nazareth (2019)
SMP_C	Non-rhizospheric sediment or soil collected in an area with vegetation	2	Ice box	Not declared	Stored at − 20 °C/ Stored at -80 °C	333	Myovela et al. (2025)
SMP_C	Non-rhizospheric sediment or soil collected in an area with vegetation	2	Ice box	Not declared	Flash-frozen in liquid nitrogen + Stored at -80 °C	338	Mahanty et al. (2024)
SMP_C	Non-rhizospheric sediment or soil collected in an area with vegetation	1	Ice box	Not declared	Not declared	368	Shah et al. (2024)
SMP_C	Non-rhizospheric sediment or soil collected in an area with vegetation	54	Not declared	Not declared	Stored at -80 °C	397	Zhang et al. (2024a)
SMP_C	Non-rhizospheric sediment or soil collected in an area with vegetation	18	Not declared- data from another unselected document	Not declared- data from another unselected document	Not declared- data from another unselected document	443	Costa et al. (2020)
SMP_D	Rhizospheric Soil	12	Not declared	(**v**) Washing (sterile water) part of the soil approximately 1 mm thick around the root	Stored at -80 °C	64	Zhou et al. (2021)
SMP_D	Rhizospheric Soil	3	Portable cooler at 4 °C	(**v**) Washing (sterile water) part of the soil approximately 1 mm thick around the root	Stored at -80 °C	77	Wang et al. (2021)
SMP_D	Rhizospheric Soil	6	Not declared	(**v**) Washing (sterile water) part of the soil approximately 1 mm thick around the root	Stored at -80 °C	91	Zhuang et al. (2020)
SMP_D	Rhizosphere soil	9	Portable refrigerator	(**vi**) liquid nitrogen cold milling	Stored at -80 °C	394	Li et al. (2024)
SMP_D	Rhizosphere soil	9	Dry ice	(**vii**) soils adhering along the secondary roots after shaking vigorously	Stored at -80 °C	448	Vanegas et al. (2019)
SMP_D	Rhizosphere soil	64	Dry ice	Not declared	Stored at − 20 °C	379	Muwawa et al. (2024)
SMP_E	parts of plants (leaves, trunks, and branches)	12	Not declared	(**viii**) homogenized in liquid nitrogen using a mortar and pestle	Liquid nitrogen	236	Arfi et al. (2012)]
SMP_E	parts of plants (root endosphere)	12	Not declared	(**ix**) Sterilization (1 min in 80% ethanol and then sterilized again for 1 min in 0.25% NaClO) + thorough grinding with liquid nitrogen	Stored at -80 °C	64	Zhou et al. (2021)
SMP_E	parts of plants (root endosphere)	3	Portable cooler at 4 °C	(**ix**) Sterilization (1 min in 80% ethanol and then sterilized again for 1 min in 0.25% NaClO) + thorough grinding with liquid nitrogen	Stored at -80 °C	77	Wang et al. (2021)
SMP_E	parts of plants (root endosphere)	6	Not declared	(**ix**) Sterilization (1 min in 80% ethanol and then sterilized again for 1 min in 0.25% NaClO) + thorough grinding with liquid nitrogen	Stored at -80 °C	91	Zhuang et al. (2020)
SMP_E	parts of plants (leaves)	48	Ice box	(**x**) TO EPIPHYTIC FUNGI: washed from leaf surfaces + sterile cooled TE buffer (10 mM Tris–HCl, 1 mM EDTA, pH 7.5) + Sonication (45 s), Vortexing (30s)+ Centrifugation (10,000×g for 10 min); TO ENDOPHYTIC FUNGI: Sterilization (immersion in 1% NaClO for 2 min, 70% EtOH for 2 min and rinsed twice in sterile, autoclaved water for 5 min) (immersion for 1 min in 75% ethanol, 3 min in 3.25% sodium hypochlorite, and 30 s in 75% ethanol, followed by three rinses in sterile distilled water + freeze-dried using liquid nitrogen and homogenized using a sterilized mortar	Stored at -80 °C	135	Yao et al. (2019)
SMP_E	parts of plants (leaves, fruits, and pneumatophores - aerial roots)	270	Not declared	(**xi**) Sterilization (1% NaClO for 2 min, 70% EtOH for 2 min, and rinsed twice in sterile, autoclaved water for 5 min) + Homogenization (Omni Bead Ruptor 24 (Omni International, Kennesaw, GA, United States at 8 m/s for 2 min)	Not declared	97	Lee et al. (2020)
SMP_E	parts of plants (leaves, fruits, and pneumatophores - aerial roots)	209	Not declared	(**xi**) Sterilization (immersion in 1% NaClO for 2 min, 70% EtOH for 2 min, and rinsed twice in sterile, autoclaved water for 5 min) + Homogenization (Omni Bead Ruptor 24 (Omni International, Kennesaw, GA, United States at 8 m/s for 2 min)	Not declared	264	Lee et al. (2019)
SMP_E	parts of plants (root episphere)	12	Not declared	(**xii**) washing and extensive shaking in 1× TE buffer supplemented with 0.1% Triton X-100 + filtration (0.22 µM)	Stored at -80 °C	64	Zhou et al. (2021)
SMP_E	parts of plants (root episphere)	3	Portable cooler at 4 °C	(**xii**) washing and extensive shaking in 1× TE buffer supplemented with 0.1% Triton X-100 + filtration (0.22 µM)	Stored at -80 °C	77	Wang et al. (2021)
SMP_E	parts of plants (root episphere)	6	Not declared	(**xii**) washing and extensive shaking in 1× TE buffer supplemented with 0.1% Triton X-100 + filtration (0.22 µM)	Stored at -80 °C	91	Zhuang et al. (2020)
SMP_E	parts of plants (leaves, roots, and stems)	8	Ice box	(**xiii**) Washed in sterile nuclease-free water and then placed in PBS for 1 h.	Stored at − 20 °C	333	Myovela et al. (2025)
SMP_E	parts of plants (leaves, roots, and stems)	3	Ice box	(**xiv**) Washed with running tap water + rinsed three times with distilled water + Sterilization (70% ethanol for 1 min, 1.0% sodium hypochlorite (NaOCl) for 1 min and further cleaned by passing through two sets of sterile distilled water OR 70% ethanol for 5 s, immersed in 4% sodium hypochlorite for 90 s and rinsed in sterile water for 10 s)	Not declared	368	Shah et al. (2024)
SMP_E	parts of plants (leaves)	36	Ice box	**(xv**) Placed in PBS + sonication + vortexing + centrifugation	Stored at -80 °C	336	Yang et al. (2024)
SMP_E	parts of plants (leaves)	17	Cool box	Not declared	Freeze drying	141	Chi et al. (2019)
SMP_F	Sediment or soil collected in non-vegetated area	3	Ice box	Not declared	Stored at -40 °C	73	Zhang et al. (2021)
SMP_F	Sediment or soil collected in non-vegetated area	2	Ice box	Not declared	Flash-frozen in liquid nitrogen + Stored at -80 °C	338	Mahanty et al. (2024)
SMP_G	Sediments kept in microcosms without vegetation and added with micro-sized polypropylene	3	Not declared	(**xvi**) Controlled addition of filtered seawater to the sediment-containing flasks, along with the addition of microplastics sterilized in an ultraclean bench for 30 min	Stored at -80 °C	380	Lin et al. (2024)

**Table 4:** Extraction of DNA and sequencing methods from sample types included in the selected fungal ITS amplicon studies in this systematic review

Reference	ID Article	Sample Type	Samples Characteristics	Number of sequenced samples totals	Method Extraction type	Method Description: Extraction of DNA	Primers	Plataform
Yu et al. (2023)	3	SMP_A	Sediments kept in microcosms without vegetation	10	Commercial kit	E. Z.N.A™ Mag-Bind Soil DNA Kit, realized by Sangon Biotech, Shanghai, China.	Data from another selected article (ID 480)- ITS1F and ITS2R	Illumina (MiSeq)
Yu et al. (2023)	3	SMP_B	Sediments were kept in microcosms without vegetation and contaminated with hydrocarbons.	12	Commercial kit	E. Z.N.A™ Mag-Bind Soil DNA Kit, realized by Sangon Biotech, Shanghai, China.	Data from another selected article (ID 480)- ITS1F and ITS2R	Illumina (MiSeq)
Zhou et al. (2021)	64	SMP_C	Non-rhizospheric sediment or soil collected in an area with vegetation	12	Commercial kit combined with the modified method	Power Soil DNA Isolation Kit + SDS	ITS1F and ITS2R	Illumina (HiSeq)
Zhou et al. (2021)	64	SMP_D	Rhizospheric Soil	12	Commercial kit combined with the modified method	Power Soil DNA Isolation Kit + SDS	ITS1F and ITS2R	Illumina (HiSeq)
Zhou et al. (2021)	64	SMP_E	parts of plants (root episphere)	12	Commercial kit	DNA PowerWater kit	ITS1F and ITS2R	Illumina (HiSeq)
Zhou et al. (2021)	64	SMP_E	parts of plants (root endosphere)	12	Commercial kit	Power Plant DNA Isolation Kit	ITS1F and ITS2R	Illumina (HiSeq)
Zhang et al. (2021)	73	SMP_C	Non-rhizospheric sediment or soil collected in an area with vegetation	107	Commercial kit	DNeasy PowerSoil Kit^®^	ITS9Munngs and ITS4ngs; ITS1F and ITS4	PacBio
Zhang et al. (2021)	73	SMP_F	Sediment or soil collected in non-vegetated area	3	Commercial kit	DNeasy PowerSoil Kit^®^	ITS9Munngs and ITS4ngs; ITS1F and ITS4	PacBio
Wang et al. (2021)	77	SMP_C	Non-rhizospheric sediment or soil collected in an area with vegetation	3	Commercial kit combined with the modified method	Power Soil DNA Isolation Kit + SDS	ITS1F and ITS2R	Illumina (HiSeq)
Wang et al. (2021)	77	SMP_D	Rhizospheric Soil	3	Commercial kit combined with the modified method	Power Soil DNA Isolation Kit + SDS	ITS1F and ITS2R	Illumina (HiSeq)
Wang et al. (2021)	77	SMP_E	parts of plants (root episphere)	3	Commercial kit	Power Water DNA Isolation Kit	ITS1F and ITS2R	Illumina (HiSeq)
Wang et al. (2021)	77	SMP_E	parts of plants (root endosphere)	3	Commercial kit	Power Plant DNA Isolation Kit	ITS1F and ITS2R	Illumina (HiSeq)
Zhuang et al. (2020)	91	SMP_D	Rhizospheric Soil	6	Commercial kit combined with the modified method	Power Soil DNA Isolation Kit + SDS	ITS1F and ITS2R	Illumina (HiSeq)
Zhuang et al. (2020)	91	SMP_C	Non-rhizospheric sediment or soil collected in an area with vegetation	6	Commercial kit combined with the modified method	Power Soil DNA Isolation Kit + SDS	ITS1F and ITS2R	Illumina (HiSeq)
Zhuang et al. (2020)	91	SMP_E	parts of plants (root episphere)	6	Commercial kit	Power Water DNA Isolation Kit	ITS1F and ITS2R	Illumina (HiSeq)
Zhuang et al. (2020)	91	SMP_E	parts of plants (root endosphere)	6	Commercial kit	Power Plant DNA Isolation Kit	ITS1F and ITS2R	Illumina (HiSeq)
Lee et al. (2020)	97	SMP_C	Non-rhizospheric sediment or soil collected in an area with vegetation	81	Commercial kit	DNeasy PowerSoil Kit	ITS1F and ITS2R	Illumina (MiSeq)
Lee et al. (2020)	97	SMP_E	parts of plants (leaves, fruits, and pneumatophores - aerial roots)	270	Commercial kit	DNeasy PowerSoil Kit	ITS1F and ITS2R	Illumina (MiSeq)
Yao et al. (2019)	135	SMP_E	parts of plants (leaves)	48	CTAB extraction method with modification.	CTAB extraction method with modification.	ITS1F and ITS2R	Illumina (MiSeq)
Chi et al. (2019)	141	SMP_E	parts of plants (leaves)	17	Commercial kit	DNeasy Plant Mini Kit	NSA3 and NLC2; ITS1-F_KYO1 and ITS2R	Illumina (MiSeq)
Cheung et al. (2018)	145	SMP_C	Non-rhizospheric sediment or soil collected in an area with vegetation	18	Commercial kit	PowerSoil DNA Isolation Kit	ITS1F and ITS2R	Ion Torrent PGM system
Arfi et al. (2012)	236	SMP_E	parts of plants (leaves, trunks, and branches)	12	Commercial Kit	NucleoSpin Plant II Kit’s DNA	ITS1F and ITS2R	454 (Life Sciences/Roche)
Lee et al. (2019)	264	SMP_C	Non-rhizospheric sediment or soil collected in an area with vegetation	70	Commercial kit	DNeasy PowerSoil Kit	ITS1F and ITS2R	Illumina (MiSeq)
Lee et al. (2019)	264	SMP_E	parts of plants (leaves, fruits, and pneumatophores - aerial roots)	209	Commercial kit	DNeasy PowerSoil Kit	ITS1F and ITS2R	Illumina (MiSeq)
Haldar and Nazareth (2019)	266	SMP_C	Non-rhizospheric sediment or soil collected in an area with vegetation	2	Not declared	Not declared	ITS1F and ITS2R	Illumina (MiSeq)
Myovela et al. (2025)	333	SMP_C	Non-rhizospheric sediment or soil collected in an area with vegetation	2	Commercial kit	ZymoBIOMICS DNA Miniprep Kit	ITS1F and ITS2R	Illumina (MiSeq)
Myovela et al. (2025)	333	SMP_E	parts of plants (leaves, roots, and stems)	8	Commercial kit	ZymoBIOMICS DNA Miniprep Kit	ITS1F and ITS2R	Illumina (MiSeq)
Yang et al. (2024)	336	SMP_E	parts of plants (leaves)	36	Commercial kit	E. Z.N.A.^®^ Soil DNA Kit	ITS1F and ITS2R	Illumina (MiSeq)
Mahanty et al. (2024)	338	SMP_C	Non-rhizospheric sediment or soil collected in an area with vegetation	2	Commercial kit	DNeasy Power SoilPro Kits	ITS1F and ITS2R	Illumina (HiSeq)
Mahanty et al. (2024)	338	SMP_F	Sediment or soil collected in non-vegetated area	2	Commercial kit	DNeasy Power SoilPro Kits	ITS1F and ITS2R	Illumina (HiSeq)
Shah et al. (2024)	368	SMP_C	Non-rhizospheric sediment or soil collected in an area with vegetation	1	Commercial kit	DNeasy Plant Pro Kit & DNeasy 96 PowerSoil Pro Kit	ITS1F and ITS2R	Illumina (MiSeq)
Shah et al. (2024)	368	SMP_E	parts of plants (leaves, roots, and stems)	3	Commercial kit	DNeasy Plant Pro Kit & DNeasy 96 PowerSoil Pro Kit	ITS1F and ITS2R	Illumina (MiSeq)
Muwawa et al. (2024)	379	SMP_D	Rhizospheric Soil	64	Commercial kit	Power Soil DNA isolation kit	ITS3tagmix1 and TS3tagmix2; ITS3tagmix3 and ITS3tagmix4; ITS3tagmix5 and ITS4ngs	Illumina (MiSeq)
Lin et al. (2024)	380	SMP_G	Sediments were maintained in microcosms without vegetation and were supplemented with micro-sized polypropylene.	3	Not declared	Not declared	ITS1F and ITS2R	Illumina (HiSeq)
Li et al. (2024)	394	SMP_D	Rhizospheric Soil	9	Commercial kit	Powerful Soil DNA Extraction Kit	ITS5 and ITS2R	Illumina (MiSeq)
Zhang et al. (2024a)	397	SMP_C	Non-rhizospheric sediment or soil collected in an area with vegetation	54	CTAB extraction method	CTAB extraction method	ITS1F and ITS2R	Illumina (NovaSeq)
Costa et al. (2020)	443	SMP_C	Non-rhizospheric sediment or soil collected in an area with vegetation	18	Not declared- data from another unselected document	Not declared- data from another unselected document	Not declared- data from another unselected document	Ion Torrent PGM
Vanegas et al. (2019)	448	SMP_D	Rhizospheric Soil	9	Commercial kit	PowerLyzer^®^PowerSoil^®^ DNA Isolation kit	ITS1F and ITS2R	Illumina (MiSeq)
Yu et al. (2022)	480	SMP_A	Sediments kept in microcosms without vegetation	SAME ARTICLE SELECTED ID 3	Commercial kit	E. Z.N.A™ Mag-Bind Soil DNA Kit, realized by Sangon Biotech, Shanghai, China.	ITS1F and ITS2R	Illumina (MiSeq)
Yu et al. (2022)	480	SMP_B	Sediments were kept in microcosms without vegetation and contaminated with hydrocarbons.	SAME ARTICLE SELECTED ID 3	Commercial kit	E. Z.N.A™ Mag-Bind Soil DNA Kit, realized by Sangon Biotech, Shanghai, China.	ITS1F and ITS2R	Illumina (MiSeq)

#### Download of raw data

After completing the systematic review, we performed a reanalysis of public ITS metabarcoding datasets using a unified bioinformatics pipeline to produce a quantitative, integrative synthesis.

Raw sequence data deposited in the NCBI Sequence Read Archive (SRA) (Leinonen et al. 2010) were retrieved with the fasterq-dump utility from the SRA Toolkit. MG-RAST (Keegan et al. 2016) and the National Genomics Data Center (NGDC)—part of the China National Center for Bioinformation (CNCB)—were downloaded manually.

Amplicon metagenomic data were downloaded from the National Center for Biotechnology Information (NCBI) SRA database with the following codes: SRR22964905 to SRR22964912 for article ID 3 (Yu et al. 2023); SRR23021236 to SRR23021236 for article ID 3 (Yu et al. 2023); SRR13255669 to SRR13255716 for article ID 64 (Zhou et al. 2021); SRR11392201 to SRR11392212 for article ID 77 (Wang et al. 2021); SRR11311104 to SRR11311127 for article ID 91 (Zhuang et al. 2020); SRR31431284 to SRR31431288, SRR31431290 to SRR31431293 for article ID 333 (Myovela et al. 2025); SRR14371793 to SRR14371856 for article ID 379 (Muwawa et al. 2024); The article ID 97 (Lee et al. 2020), 151 SRA codes were downloaded, but 114 codes were excluded in the data filtering stage, because they had only 0 or 1 number of reads in all taxa, so only 37 codes were used for metagenomic analysis (**Supplementary Material **6**).**

Additional data was obtained from two other databases. Information from the National Genomics Data Center (NGDC) was downloaded using the codes CRR1110770 to CRR1110787, referring to article ID 336 (Yang et al. 2024). In addition, other data were retrieved from the Metagenomics Rapid Annotation using Subsystem Technology (MG-RAST) database, with the following codes: mgm4826250.3, mgm4826252.3, mgm4826254.3, mgm4826255.3, mgm4826256.3, mgm4826258.3, mgm4826259.3, mgm4826260.3, mgm4826261.3, mgm4826264.3, mgm4826265.3, mgm4826266.3, referring to article ID 448 (Vanegas et al. 2019). The codes mgm4826251.3, mgm4826253.3, mgm4826257.3, mgm4826262.3, and mgm4826267.3 were excluded in the data filtering stage, because they had only 0 number of reads in all taxa.

#### Taxonomic annotation of raw data

The taxonomic annotations for each data group (per article) were performed using the ITS amplicon analysis pipeline, the source code of which is available on GitHub: https://github.com/LBMCF/pipeline-for-amplicon-analysis. The pipeline enabled analysis of amplicon reads sequenced on Illumina platforms and returned a table of taxonomic abundances, with predicted lineages based on UNITE 10.0 (Abarenkov et al. 2024) for ITS. The pipeline integrated the programs: 32-bit USEARCH (Edgar 2010), VSEARCH (Rognes et al. 2016), Cutadapt (Martin 2011), and FastQC (Andrews 2010).

Initially, the quality control of the reads was evaluated using FastQC, were merged all the direct and reverse readings using VSEARCH with the --fastq_mergepairs option, cut the adapters using Cutadapt, quality control using VSEARCH with the --fastq_filter options, duplicates were eliminated using VSEARCH with the --derep_fulllength option, the taxonomic table was generated using USEARCH with the --unoise3 option, the abundance table was generated using the --usearch_global and id = 0 options. 99, the taxonomic classifications were performed using the SINTAX algorithm (Edgar 2016) in USEARCH and the UNITE taxonomic database (Abarenkov et al. 2024). Finally, the abundance table was merged with the taxonomic classifications using the local script get_abundances_table_asv.py, which is included in the source code. Once the taxonomic annotations were completed, the results were compiled using a local Python script. Taxonomy was assigned to the representative Taxon for each sample, excluding samples with read counts < 1% in the sequencing data (Data Filtration). This table was exported as a CSV file for use in R until the subsequent analysis is conducted.

#### Analysis of fungal communities obtained from mangrove substrates

Fungal community analyses of mangrove samples were performed using R v4.2.1. After data extraction, absolute and relative frequencies were calculated for each phylum and genus of the samples and analyzed using bar charts generated with the ggplot2 v3.4.3 package (Wickham 2011). For analysis of shareability and exclusivity patterns and alpha and beta diversity, taxonomic data obtained as unclassified were excluded, and only data classified at the genus level were used for these analyses. To compare shareability and uniqueness patterns of fungal taxa among distinct substrata, Venn diagrams were built with the Venn v1.10 R package (Dusa 2017). The patterns of sharing and exclusivity of fungal genera from three distinct groups were also analyzed: (1) macrofungi collected and/or isolated from cultures, (2) microfungi isolated from cultures, and (3) genera identified using ITS region metabarcoding techniques. Data on macrofungal and microfungal isolates were retrieved from public databases, such as NCBI, including not only the names of the genera, but also the names of the strains, substrate types, and the location (geographical regions) to which the mangrove belonged (**Supplementary Material **7). These data were analyzed using Venn diagrams constructed with Venny 2.1.

Alpha diversity was carried out using the Shannon diversity index using the vegan v2.6.4 package (Dixon 2003) and plotted with ggplot2 v3.4.3, based on relative abundance at the genus level. Beta Diversity was evaluated using Principal Coordinates Analysis, with the packages vegan v2.6.4, ggrepel v0.9.3 (Slowikowski et al. 2018), and ggplot2 v3.4.3. Differences in OTU composition (alpha and beta diversity) among substrates were evaluated with permutational multivariate analysis of variance (PERMANOVA; Anderson 2001).

#### Ecological traits of fungal taxa

To evaluate the ecological traits of fungal genera, we used the FungalTraits database (Põlme et al. 2020) as a reference. OTUs identified at the genus level were compared against the database, and, in case of a match, trait information was annotated in each respective OTU. Only OTUs identified at the genus level were annotated. Genera absent in the FungalTraits database were annotated as “unclassified.” All subsequent data analysis, including the calculation of the percentage of OTUs or genera identified, the relative abundance (%) of fungal genera per FungalTraits, and the generation of all figures, was performed using the Python programming language within the Google Colaboratory environment.

## Results

### Identification and selection of studies

Potentially eligible records were identified in the three selected databases: PubMed (*n* = 333), Scopus (*n* = 358), and Web of Science (*n* = 454), totaling 1145 records (**Supplementary Material **3**)**. Among these records, 666 duplicate records were automatically removed before the initial screening, resulting in 479 documents. In the screening, nine documents without a DOI, two unavailable articles, and 30 other types of documents, such as book chapters (*n* = 6), book (*n* = 1), review articles (*n* = 19), and systematic review articles (*n* = 4), were removed, resulting in 438 original articles. After the authors had read the titles and abstracts of the 438 original articles on the website https://rayyan.ai, eight articles were initially selected. Subsequently, 417 articles were excluded, and 13 were classified as ‘maybe’ for further evaluation. Subsequently, the 438 original articles were downloaded as PDFs and thoroughly reviewed, with particular attention to the materials and methods and results sections. After this second analysis, the eight selected articles were maintained (out of the 417 articles that had been excluded), two were recovered and selected, and of the 13 articles that had been classified as ‘maybe’, 12 articles were selected in full, and were verified that they met the inclusion criteria listed in Table 1.

**Fig. 1:**
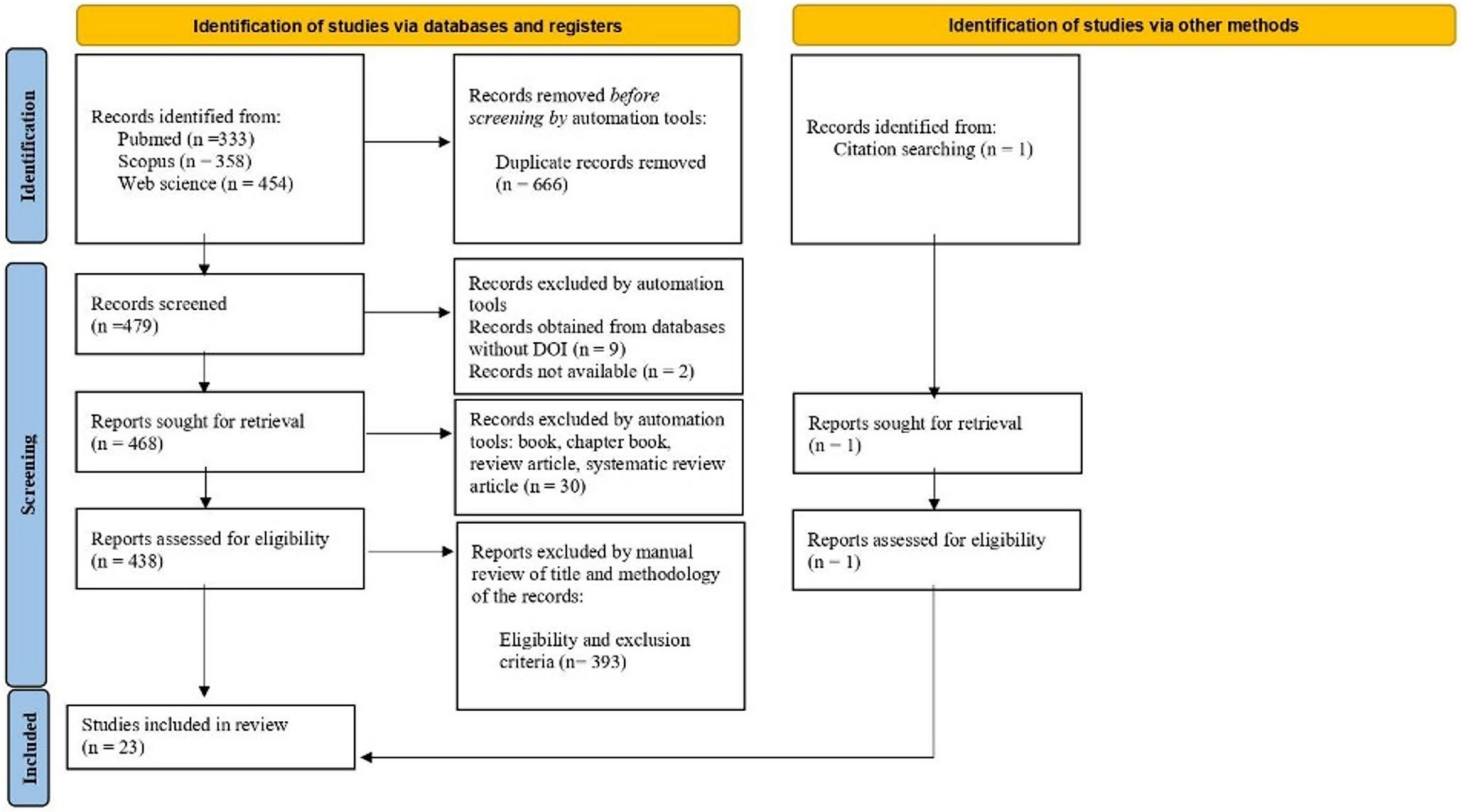
PRISMA Flowchart for this systematic review

The 438 original articles were evaluated using the inclusion and exclusion criteria described in Table 1, based on the collection site, sample type, microorganism type, sequencing method, and microorganism genomic region. Following these inclusion and exclusion criteria, 416 articles were excluded after complete reading, for four specific reasons: Reason 1: Other ecosystems or locality, nominal characteristics from mangroves not made explicit; Reason 2: culture-dependent molecular methods under standard laboratory conditions; Reason 3: Different type of sequencing (shotgun, metatranscriptomic, genomic, transcriptomic) or amplicon region other than ITS; Reason 4: Without molecular methods for analyzing the microbiome. One article not found in the data search was also selected as a relevant study for this review. It was identified from the bibliographic reference of another previously selected article. Therefore, 23 original articles from the screening phase and assessment of compliance with the inclusion or exclusion criteria were selected for synthesis and data analysis in this systematic review (Fig. 1; **Supplementary Material **4).

### Included studies

#### Publications per year

The included studies were published between 2012 and 2025 (Table 2). These studies evaluated the mangrove microbiome using DNA sequencing analyses of non-rhizospheric and rhizospheric sediments or soil collected from vegetated and non-vegetated areas, as well as from plant organs (roots, stems, leaves, and fruits). In 2012, 2018, 2022, 2023, and 2025, only one article was published per year. In contrast, 2024 and 2019 had seven and five articles per year, respectively, whereas 2020 and 2021 had only three articles per year (Table 2).

#### Journal overview of included studies

The 23 articles selected for this systematic review were published in 18 peer-reviewed scientific journals, reflecting a multidisciplinary focus on fungal communities in environmental contexts. The journals cover areas such as fungal biology, biotechnology, environmental sciences, microbial ecology, and microbiology. Among these, the journals Frontiers in Microbiology, Journal of Hazardous Materials, Microbiome, and Scientific Reports were notable for each contributing two articles to the included dataset. The other 14 journals (*n* = 15) were represented by only one selected article.

#### Geographic region and sampling

**Fig. 2:**
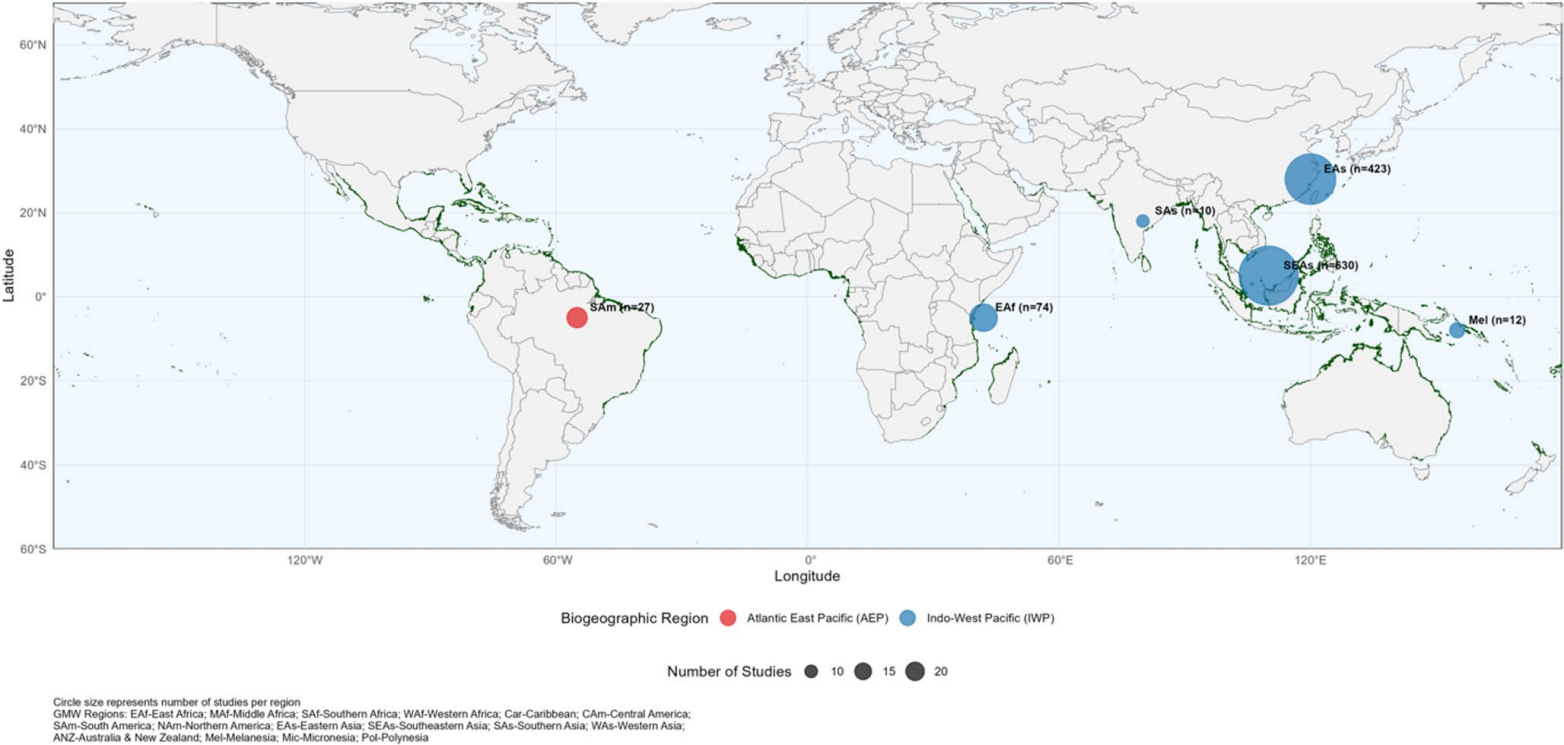
Distribution of mangrove geographic regions on the world map with the number of samples for each region. Green lines in coastal areas indicate where mangrove forests occur

The selected studies reported the taxonomic classification and relative abundance of the fungal community (sequenced using amplicon metagenomics of the ITS region) across various independent culture samples obtained from mangroves in three distinct geographic areas. The geographic distribution of the mangroves from which the analyzed samples were collected is shown in Fig. 2. During the data selection for this review, it was noted that articles ID 3 and ID 480 are complementary publications derived from the same study and that both articles refer to the same set of sequenced samples and display overlapping data. Therefore, they were considered as a single study to avoid duplication in the stages of sampling and analysis of fungal taxonomic data.

Among the 23 articles selected, a total of 1154 samples was analyzed. The highest number of samples (*n* = 630; 54.59%) came from the **Southeastern Asia region- SEAs**, followed by **Eastern Asia-EAs** (*n* = 401; 34.75%), **East Africa - EAf** (*n* = 74; 6.41%), **South America- SAm** (*n* = 27; 2.34%), **Melanesia- Mel** (*n* = 12; 1.04%) and **Southern Asia- SAs** (*n* = 10; 0.87%).

The studies evaluated samples collected from mangroves located in China and Taiwan (**EAs**), Brazil and Colombia (**SAm**), Malaysia and Singapore (**SEAs**), India (**SAs**), Tanzania and Kenya (**EAf)**, and New Caledonia (**Mel**) **(**Table 2**)**. The most of these studies (*n* = 384; 33.27%) were carried out with samples from China mangroves, followed by Singapore (*n* = 329; 28.51%), Malaysia (*n* = 301; 26.08%), Kenya (*n* = 64; 5.55%); Brazil (*n* = 18; 1.56%); Taiwan (*n* = 17; 1.47%), New Caledonia (*n* = 12; 1.04%), India (*n* = 10; 0.87%), Tanzania (*n* = 10; 0.87%) and Colombia (*n* = 9; 0.78%) **(**Table 2**).**

Among the seven identified sample types and the 1154 samples analyzed by metagenomic amplicon sequencing of the ITS region, the most frequently substrate were parts of the native and associated mangrove plant species (**SMP_E**), such as roots, stems/trunks, leaves, flowers and fruits, totaling 645 samples and representing 55.89% of all samples analyzed **(**Fig. 2**and** Table 2**).** The second most frequently examined substrate was non-rhizospheric sediment/soil collected in an area with vegetation (**SMP_C)**, comprising 376 samples (32.58%). The other types of samples were analyzed in smaller proportions: Rhizospheric Soil **(SMP_D)** examined 103 samples (8.92%), followed by sediments kept in microcosms without vegetation and contaminated with hydrocarbons (**SMP_B**) with 12 samples (1.04%), sediments kept in microcosms without vegetation (**SMP_A**) with 10 samples (0.87%), sediment or soil collected in non-vegetated area (**SMP_F**) with 5 samples (0.43%), and sediments kept in microcosms without vegetation and added with micro-sized Polypropylene (**SMP_G**) with only 3 samples (0.26%) **(**Fig. 2**and** Table 2**).**

The sample (**SMP_G**) was obtained from a single geographical mangrove region (**EAs -** China) and corresponded to a single article among the 12 selected. The samples **SMP_A** and **SMP_B** were also obtained from a geographical region (**EAs-** China) but were described in two articles that complement each other. In comparison, the **SMP_C** and **SMP_E** samples were collected extensively across five distinct mangrove regions, demonstrating a broad geographic range, and were reported in 13 and 11 articles, respectively. Nonetheless, the specific areas for each type of these substrates were different. The **SMP_C** samples were obtained from regions **EAs** (China), **SAm** (Brazil), **SEAs** (Malaysia/Singapore), **SAs** (India), and **EAf** (Tanzania). In contrast, the **SMP_E** samples were collected in the **EAs** (China/Taiwan), **SEAs** (Malaysia/Singapore), **SAs** (India), **EAf** (Tanzania), and **Mel** (New Caledonia). Finally, SMP_D samples were obtained from three geographical regions (**EAs** - China, **SAm** - Colombia, **EAf** - Kenya) and appeared in six articles **(**Fig. 2; Table 2, **and Supplementary Material **5**).**

### Methods of preprocessing, preservation, and extraction of DNA from selected sample types

This section presents the methods used for sample preprocessing, preservation, and DNA extraction in studies focusing on various mangrove substrates (**SMP_A**,** SMP_B**,** SMP_C**,** SMP_D**,** SMP_E**,** SMP_F**, and **SMP_G)**,** as** shown in Table 3.

#### Sample storage methods for DNA extraction at the collection site

Three hundred forty-one (341) samples (from twelve articles)— 133 **SMP_C**, **85 SMP_D**, 118 **SMP_E**, and 5 **SMP_F**—were explicitly mentioned as being stored in cold conditions (including ice box, cool box, dry ice, portable cooler at 4 °C, and portable refrigerator) at the time of collection. In contrast, 813 samples lacked details regarding storage conditions at the collection site. The subsequent steps, involving preprocessing methods and storage in the laboratory, varied across studies (Table 3).

#### DNA preprocessing methods

The selected studies employed different approaches to sample preparation before DNA extraction. These approaches involved physical and chemical treatments designed to remove contaminants, reduce microbial load on external surfaces, or homogenize the material for efficient downstream processing. The sample pre-processing protocols were diverse and adapted to the different types of substrates and study objectives. Below is a consolidated overview of the main protocols (Table 3):

For sediment microcosm samples, protocol **(i)** involved incubating samples in uncontaminated (**SMP_A**) or hydrocarbon-contaminated (HBCDs- **SMP_B**) microcosms at room temperature (25 °C) for up to 20 months. After this period, the samples were collected and homogenized. This method was detailed in two complementary articles (ID 3 and ID 480), covering 10 **SMP_A** samples and 12 **SMP_B** samples; For non-rhizosphere sediment samples collected in vegetated areas (**SMP_C**), protocol (**ii**) described homogenization using an Omni Bead Ruptor 24, a mechanical disruption approach that ensures sediment uniformity by breaking up larger aggregates, as reported in two papers (ID 97 and ID 264) for 151 **SMP_C** samples. Additionally, protocol (**iii**) employed sieving through a 2 mm mesh for 18 **SMP_C** samples (ID 145 article), a crucial step to remove larger debris, stones, or roots, resulting in a finer sediment fraction more suitable for consistent DNA extraction. A third method for **SMP_C** (**iv**) consisted of shaking the root to remove loosely attached soil, presumably to recover non-rhizospheric sediment, as described in three articles (ID 64, ID 77, ID 91) for 21 **SMP_C** samples. For the rhizospheric soil samples (**SMP_D**), protocol (**v**) used to wash only with sterile water as a gentle method to clean debris from the rhizospheric soil collected at approximately 1 mm thickness around the root, a procedure present in three articles (ID 64, ID 77, ID 91) for 21 **SMP_D** samples. Protocol (**vi**) also described cold grinding with liquid nitrogen for 9 **SMP_D** samples in one article (ID 394). In addition, in one article (ID 448), protocol (**vii**) mentioned the process of vigorous shaking to remove soils adhered along secondary roots for 9 **SMP_D** samples; Various protocols were applied to the plant part samples (**SMP_E**), including leaves, stems, roots, trunks, branches, pneumatophores- aerial roots and fruit: Protocol **(viii)** described of homogenized of samples in liquid nitrogen using a mortar and pestle (ID 236 − 12 **SMP_E** samples**)**; Protocol (**ix**) involved sterilization steps and complete grinding with liquid nitrogen for 21 **SMP_E** samples (root endosphere) as reported in three articles (ID 64, ID 77 and ID 91). Sterilization protocols included immersion in 80% ethanol and 0.25% NaClO to eliminate surface contaminants on plant parts, and grinding in liquid nitrogen to ensure effective cell rupture, particularly for resistant plant tissues. For epiphytic and endophytic fungi, protocol (**x**) described a multifaceted process for 48 **SMP_E** samples in one article (ID 135). For epiphytic fungi on leaf surfaces, the method included washing the leaf surfaces, using sterile and cooled TE buffer (10 mM Tris-HCl, 1 mM EDTA, pH 7.5), sonication (45 s), vortexing (30 s) and centrifugation (10,000×g for 10 min), followed by sequential sterilization with multiple immersions in NaClO and ethanol, interspersed with water. For endophytic fungi, the protocol combined sterilization (immersion in 1% NaClO, 70% ethanol, and multiple rinses), freeze-drying with liquid nitrogen, and homogenization with a sterile mortar. Both processes aimed to ensure mechanical disruption and robust surface decontamination. Protocol (**xi**) applied sterilization (1% NaClO, 70% ethanol, autoclaved water) followed by homogenization (Omni Bead Ruptor 24) to 479 SMP_E samples (healthy leaves, fruits, and pneumatophores- aerial roots), combining chemical decontamination with physical disruption, as described in two articles (ID 97 and ID 264). For root episphere (**SMP_E**), the protocol (**xii**) detailed washing with extensive agitation, supplementation with TE buffer and 0.1% Triton X-100, and filtration (0.22 μm) for 21 SMP_E samples in articles (ID 64, ID 77, ID 91), to remove finer particles and microbial cells not strongly associated with plant tissues. In the case of stems, roots and leaves (**SMP_E**), two protocols were identified: protocol (**xiii**) involved washing in sterile nuclease-free water and placing in PBS for 1 h for 8 SMP_E samples in one article (ID 333); and protocol (**xiv**) described washing with running tap water and three rinses with distilled water, followed by sterilization (with variations between 70% ethanol, 1.0% NaClO and washing with sterile distilled water OR 70% ethanol, 4% sodium hypochlorite and sterile rinsing), applied to 3 **SMP_E** samples in one article (ID 368). Protocol **(xv**) specified placing in PBS, followed by sonication, vortexing, and centrifugation for 36 **SMP_E** samples (healthy leaves), as reported in an article (ID 336). Finally, for the sediment microcosm samples (**SMP_G**), protocol (**xvi**) detailed the controlled addition of filtered seawater, along with the addition of microplastics that were sterilized on an ultraclean bench for 30 min, a method found in an article (ID 380) for 3 **SMP_G** samples.

Moreover, in nine articles (272 total samples, distributed as **SMP_C**: 186, **SMP_D**: 64, **SMP_E**: 17, and **SMP_F**: 5), the preprocessing method was not declared, leaving uncertainty about the exact procedures used to prepare these samples for DNA extraction (Table 3).

#### Sample storage methods for DNA extraction in the laboratory / DNA preservation agent

After preprocessing, samples were stored using various methods to preserve DNA integrity before extraction. The following outlines the primary storage conditions reported (Table 3):

− 20 °C storage was noted in three articles, encompassing 74 samples (SMP_C: 2, SMP_D: 64, and SMP_E: 8;
− 40 °C storage was used in one article, covering 110 samples (107 SMP_C and 3 SMP_F);
−80 °C storage was the most common low-temperature approach, reported by ten articles and applied to 261 samples (93 SMP_C, 39 SMP_D, 126 SMP_E, and 3 SMP_G);
− 80 °C/−20 °C was utilized in one article, covering 2 SMP_C samples;
- Freeze drying was used in one article and applied to 17 SMP_E samples;
- Liquid nitrogen was mentioned in one article, specifically for 12 SMP_E samples, which enables ultra-cold preservation immediately after collection or preprocessing;
- Flash-frozen in liquid nitrogen, followed by -80 °C storage, was used in one article, encompassing four samples (2 SMP_C and 2 SMP_F).

Finally, five articles that encompassed 674 samples did not report their storage method, making it difficult to assess the potential impact of storage conditions on DNA integrity. Overall, these varied preprocessing, sterilization, and storage strategies reflect the methodological diversity inherent in the study of mangrove-associated samples. The choice of method often hinges on the specific research question, target substrate, and downstream molecular analysis techniques.

### DNA extraction methods

DNA extraction for amplicon-based (ITS) metagenomics was primarily conducted using three approaches: (1) commercial kits, (2) a commercial kit combined with a modified SDS method, and (3) the CTAB method **(**Table 4). Among the articles reviewed, three studies (ID 64, ID 77, and ID 91) employed both the commercial kit and the commercial kit combined with the modified SDS method, while fifteen articles used only the commercial kit. Two articles used the CTAB method (Table 4), and three did not specify the DNA extraction method.

In total, 1154 samples were subjected to DNA extraction. Among these, 102 samples used the CTAB method, including 54 SMP_C and 48 SMP_E, whereas 42 samples— 21 from **SMP_C and** 21 from **SMP_D**—were processed with the commercial kit combined with the modified SDS method. The remaining 987 samples were extracted with commercial kits, including **10 SMP_A** samples, **12 SMP_B** samples, **281 SMP_C** samples, **82 SMP_D** samples, **597 SMP_E samples**, and **5 SMP_F** samples. Overall, commercial kits were the most frequently used method, whereas the CTAB and combined kit-SDS approaches were adopted in fewer instances. Finally, 23 samples did not specify the DNA extraction method (Table 4).

### DNA amplification methods

DNA amplification for fungal internal transcribed spacer (ITS) regions was performed using nine specific primer pairs: (1): ITS1F and ITS2R; (2) ITS5 and ITS2R ; (3) ITS9Munngs and ITS4ngs; (4) ITS1F and ITS4; (5) NSA3 and NLC2; (6) ITS1-F_KYO1 and ITS2R; (7) ITS3tagmix1 and TS3tagmix2; (8) ITS3tagmix3 and ITS3tagmix4; (9) ITS3tagmix5 and ITS4ngs.

The ITS1F (CTTGGTCATTTAGAGGAAGTAA) and ITS2R (GCTGCGTTCTTCATCGATGC) primer pairs were the most widely adopted primer pairs for Amplicon Metagenomics of the ITS region, being used in 18 articles and totaling 936 sequenced samples (SMP_A: 10, SMP_B: 12, SMP_C: 251, SMP_D: 30, SMP_E: 628, SMP_F: 2, and SMP_G: 3). In contrast, the ITS5 (and ITS2R primer pairs were only used in 1 single article and for the sequencing of 9 samples (SMP_D). Three articles used combinations of more than one different pair of primers: one used two pairs of primers (NSA3/NLC2 and ITS1-F_KYO1/ITS2R) and 17 sequenced samples (SMP_E), another with two pairs of primers (ITS9Munngs/ITS4ngs and ITS1F/ITS4) and 110 samples (SMP_C: 107 and SMP_F: 3) and a complex set of primers (3 pairs-ITS3tagmix1 and TS3tagmix2; ITS3tagmix3 and ITS3tagmix4; ITS3tagmix5 and ITS4ngs ) with 64 sequenced samples (SMP_D).

### Methods of high-performance DNA sequencing

Next-generation sequencing (NGS) platforms were used to sequence the amplicons, employing both short- and long-read methods. Four platforms were used: Illumina, Ion Torrent PGM, 454 (Life Sciences/Roche), and the long-read PacBio system. Of the reviewed articles, nineteen focused on Illumina platforms, specifically MiSeq (13 articles), HiSeq (5 articles), and NovaSeq (1 article), covering 996 samples. These samples comprised 10 from SMP_A (MiSeq), 10 from SMP_B (MiSeq), 233 from SMP_C (156 with MiSeq, 54 with NovaSeq, and 23 with HiSeq), 103 from SMP_D (82 with MiSeq and 21 with HiSeq), 633 from SMP_E ( 591 with MiSeq and 42 with HiSeq), two from SMP_F (HiSeq), and three from SMP_G (HiSeq). Two articles, covering 36 SMP_C samples, employed Ion Torrent PGM, while another, analyzing 12 SMP_E samples, utilized the 454 platform. Finally, a study comprising 110 samples (107 from SMP_C and 3 from SMP_F) used the PacBio system for long-read sequencing (Table 4).

#### Physicochemical features

The tested samples were preserved at the collection site. The most used method (74.48% of samples (213), SMP_C: 126, SMP_D: 82; SMP_F: 5) was preservation in cold conditions (including ice box, dry ice, and portable refrigerator), which was reported in 7 articles and not declared (3 articles, SMP_C: 69 and SMP_G: 3), and in situ (1 article and 1 sample (water)). Some samples required preprocessing before analysis.

The sample pre-processing protocols for analyzing physicochemical characteristics were diverse and adapted to the different types of samples, such as: For non-rhizospheric sediment samples collected in vegetated areas (**SMP_C**), protocol **(1)** consisted of shaking the root to remove loosely attached soil, presumably to recover non-rhizospheric sediment, described in one article (ID 64) for 12 **SMP_**C samples; **(2)** air drying, followed by milling to obtain a fine powder and filtering through a mesh of 100 holes per inch. The processed samples are then weighed for analysis of TP, TS, TC, TN and TOC, while other parameters are analyzed without prior processing, described in one article (ID73) for 106 **SMP_**C samples and 3 **SMP_F** samples; **(3)** sieving (2 mm) + DI water mixture (1:1 v/v), to analyze pH and redox potential or **(4)** sieving (2 mm) + drying at 105 °C, to analyze total carbon, nitrogen and sulfur, described in a single article (ID 145) for 18 **SMP_C** samples; (**5**) a combination of air drying, oven drying at 60 °C, milling and sieving (75 μm), described in one article (ID338) for 2 **SMP_C** samples and 2 **SMP_F** samples; For rhizosphere soil, only one method was utilized, by **(6)** vigorous shaking to separate the adhering soil and the plant’s secondary roots (ID 448; 9 **SMP_D** samples); **(7)** combination of controlled addition of filtered seawater to flasks containing sediment, addition of microplastics, previously sterilized (30 min) on an ultraclean bench and incubation for 28 days at room temperature, described in an article (ID 380) for 3 **SMP_G** samples (Table 5). Finally, in four articles (130 total samples; distributed as **SMP_C**: 57, **SMP_D**: 73), the preprocessing method was not specified, and one article (ID 236) analyzed one water sample in situ (Table 5).

**Table 5:** Methods of preprocessing and preservation from sample types for physicochemical features or hydrocarbons concentrations (HBCDS)

Sample Type	Samples Characteristics	Number of samples analyzed	Methods for preserving samples at the collection site	Preprocessing Methods	Methods for storing sample analysis in the laboratory	ID Article	Reference
SMP_A	Sediments kept in microcosms without vegetation	18	Not declared	Microcosm of sediment without contaminated + freeze-dried and extracted with a mixture of acetone, dichloromethane, and n-hexane (1:1:1). Crude extractions were dried, and redissolved in methyl alcohol+ analysis by LC-MS+	lyophilized sediments before analysis	3 and 480 (complementary articles)	Yu et al. (2023) and Yu et al. (2022)
SMP_B	Sediments kept in microcosms without vegetation and contaminated with hydrocarbons	18	Not declared	Microcosm of sediment contaminated with HBCDS at room temperature for 20 months + freeze-dried and extracted with a mixture of acetone, dichloromethane, and n-hexane (1:1:1). Crude extractions were dried, redissolved in methyl alcohol, and analyzed by LC-MS	lyophilized sediments before analysis	3 and 480 (complementary articles)	Yu et al. (2023) and Yu et al. (2022)
SMP_C	Non-rhizosphere sediment or soil collected in an area with vegetation	12	Not declared	**(1)** Non-rhizosphere soil was shaken off the root	Not declared	64	Zhou et al. (2021)
SMP_C	Non-rhizosphere sediment or soil collected in an area with vegetation	106	Ice box	**(2)** Air-dried + ground into powder + filtered (100 holes per inch) + weighed (for TP, TS, TC, TN, and TOC), other parameters no processing carried out	Stored at − 40 °C	73	Zhang et al. (2021)
SMP_F	Sediment or soil collected in non-vegetated area	3	Ice box	**(2)** Air-dried + ground into powder + filtered (100 holes per inch) + weighed (for TP, TS, TC, TN, and TOC), other parameters no processing carried out	Stored at − 40 °C	73	Zhang et al. (2021)
SMP_C	Non-rhizosphere sediment or soil collected in an area with vegetation	18	Ice box	**(3)** sieved through a 2-mm mesh + added to 10 ml DI water (1:1 v/v) and mixed to create a slurry (measured pH and redox potential); **(4)** sieved through a 2-mm mesh + dried at 105 °C overnight (measured total carbon (TC), total nitrogen (TN), and total sulphur (TS) content)	Not declared	145	Cheung et al. (2018)
WT	Water	1	In situ	In situ	In situ	236	Arfi et al. (2012)
SMP_C	Non-rhizosphere sediment or soil collected in an area with vegetation	2	Ice box	**(5)** air-dried and then further dried in an oven, maintaining a consistent temperature of 60 °C + ground to a fine powder, and sieved through a 75 μm sieve	Not declared	338	Mahanty et al. (2024)
SMP_F	Sediment or soil collected in non-vegetated area	2	Ice box	**(5)** air-dried and then further dried in an oven, maintaining a consistent temperature of 60 °C + ground to a fine powder, and sieved through a 75 μm sieve	Not declared	338	Mahanty et al. (2024)
SMP_D	Rhizospheric Soil	64	Dry Ice	Not declared	Stored at − 20 °C	379	Muwawa et al. (2024)
SMP_C	Non-rhizosphere sediment or soil collected in an area with vegetation	3	Not declared	Not declared	Stored at 4 °C	380	Lin et al. (2024)
SMP_D	Rhizospheric Soil	9	Dry ice	**(6)** soils adhering along the secondary roots after shaking vigorously	Stored at -80 °C	448	Vanegas et al. (2019)
SMP_G	Sediments kept in microcosms without vegetation added with micro-sized Polypropylene)	3	Not declared	**(7)** Controlled addition of filtered seawater to the sediment-containing flasks, along with the addition of microplastics sterilized in an ultraclean bench for 30 min. + incubated for 28 days in 250 mL brown glass jars at room temperature.	Not declared	380	Lin et al. (2024)
SMP_D	Rhizospheric Soil	9	Portable refrigerator	Not declared	Stored at 4 °C	394	Li et al. (2024)
SMP_C	Non-rhizosphere sediment or soil collected in an area with vegetation	54	Not declared	Not declared	Stored at 4 °C	397	Zhang et al. (2024a)

Before laboratory analysis, samples were kept at **-40 °C** (109 samples, **SMP_C** (106 samples), and **SMP_F** (3 samples), kept at **-20 °C** (64 **SMP_D** samples), kept at **-80 °C** (9 **SMP_D** samples), stored at 4 °C (57 **SMP_C** samples), In situ (1 sample - WT) and not declared (19 samples (32 **SMP_C**, 2 **SMP_F** and 3 **SMP_G**) (Table 5).

Among the 23 selected articles, 10 analyzed physicochemical features (**Supplementary Material **8). Two hundred eighty-six (286) samples were tested with physicochemical analyses from the 10 articles, which were distributed as follows: SMP_C (195 samples), SMP_D (82 samples), SMP_F (5 samples), SMP_G (3 samples), and Water (WT) (1 sample) (Table 6).

**Table 6:** Frequency of measurement of physical-chemical parameters and hydrocarbon concentration in 286 mangrove samples

Parameters	Total samples analyzed	Percentage of samples analyzed (%)	Number of sample types analyzed	Number of Articles
HBCDS	36	12.59%	SMP_A: 18 and SMP_B: 18	1
Total Carbon (TC)	278	97,20%	SMP_C: 193, SMP_D: 82, SMP_F: 3	8
Total Nitrogen (TN)	275	96,15%	SMP_C: 190, SMP_D: 82, SMP_F: 3	**7**
pH	274	95,80%	SMP_C: 195, SMP_D: 73, SMP_F: 5, WT: 1	9
Salinity	271	94,76%	SMP_C: 192, SMP_D: 73, SMP_F: 5, WT: 1	8
Total Sulfur (TS)	181	63,29%	SMP_C: 178, SMP_F: 3	3
Total Organic Carbon (TOC)	179	62,59%	SMP_C: 162, SMP_D: 9, SMP_F: 5, SMP_G: 3	5
Total Phosphorus (TP)	118	41,26%	SMP_C: 106, SMP_D: 9, SMP_F: 3	2
Electrical Conductivity (EC)	107	37,41%	SMP_C: 32, SMP_D: 73, SMP_F: 2	5
Heavy metals and other elements	77	26,92%	Supplementary Material Table S6	Supplementary Material Table S6

Among the 286 samples tested, Total Carbon (TC) was the most commonly measured parameter, assessed in approximately 97.20% (278 samples; SMP_C: 193, SMP_D: 82, SMP_F: 3; 8 articles). In addition to TC, other parameters have been widely investigated, reflecting the environmental complexity of mangrove ecosystems: Total Nitrogen (TN) measured of 96.15% (275 samples, SMP_C: 190, SMP_D: 82, SMP_F: 3, 7 articles); pH with 95.80% (274 samples, SMP_C: 195, SMP_D: 73, SMP_F: 5 and water: 1; 9 articles); Salinity with 94.76% (271 samples, SMP_C: 192, SMP_D: 73, SMP_F: 5 and water: 1, 8 articles); Total Sulfur (TS) with 63.29% (181 samples, SMP_C: 178 and SMP_F: 3, 3 articles); Total Organic Carbon (TOC) with 62.59% (179 samples, SMP_C: 162, SMP_D: 9, SMP_F: 5 and SMP_G: 3, 5 articles); Total Phosphorus (TP) with 41.26% (118 samples, SMP_C: 106, SMP_D: 9, SMP_F: 3, 2 articles) and Electrical Conductivity (EC) with 37.41% (107 samples, SMP_C: 32, SMP_D: 73, SMP_F: 2, 5 articles) (Table 6).

Heavy metals and other elements (e.g., Aluminum, Cadmium, Cobalt, Chromium, Copper, Iron, Manganese, Nickel, Lead, Zinc, Boron, Calcium, Potassium, Magnesium, Sodium), important for understanding pollution and geochemical cycling, were investigated in roughly 77 samples (26.92%) (Table 6). The other physical and chemical analyses, which have received less study, are presented in **Supplementary Material **8**.**

#### Hydrocarbons (HBCDS)

Among the selected files, only two articles presented HBCDS analysis in their methodologies, with a total of 36 samples (18 SMP_A and 18 SMP_B) (Table 6 and **Supplementary Material **8). For the 18 SMP_B samples, the following were quantified: Total HBCDs (µg/mL), α-HBCD (µg/mL), β-HBCD (µg/mL), and γ-HBCD (µg/mL). The SMP_A samples (*n* = 18) were quantitatively analyzed for HBCDS amendment (µg/mL). Methods for preserving samples at the collection site and for laboratory analysis of HBCDS were not specified (Table 5). Before analysis, the samples were freeze-dried and then extracted with acetone, dichloromethane, and n-hexane (1:1:1). The crude extracts were dried and redissolved in methyl alcohol. The suspension was used for determination by Liquid Chromatograph Mass Spectrometer (LC-MS, Thermo TSQ-Endura, USA) equipped with a dual-mode discrete-dynode detector and a Hypersil GOLD 1.9 µ column (50 × 2.1 mm) (Table 5).

### Overview of taxonomic diversity

#### Richness and relative abundance of fungi in distinct mangrove substrates

After excluding all the zero or singletons, a total of 763 fungal taxa (OTU/ASV) were retrieved in 239 samples of mangrove substrates (SMP_A: 10; SMP_B: 12; SMP_C: 31; SMP_D: 91 and SMP_E: 95) and those taxa were distributed in 11 phyla, 38 classes, 102 orders, 241 families, 377 genera, and 344 putative species (**Supplementary Material** 9).

**Fig. 3:**
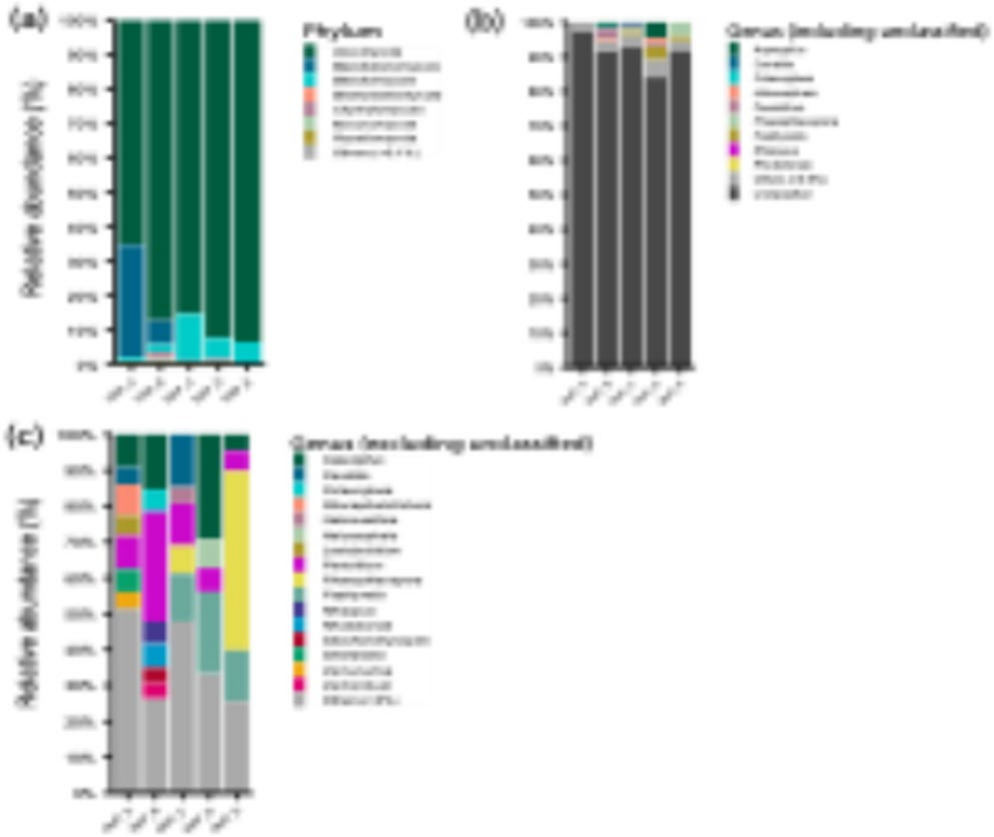
Fungal richness and relative abundance in distinct mangrove substrates. (a) Phylum; (b) Genera (including those taxa that cannot be identified at genus level); (c) Genera (only the most abundant ones, excluding those taxa that cannot be identified at genus level). Sample type: (SMP_A: Sediments kept in microcosms without vegetation; SMP_B: Sediments kept in microcosms without vegetation and contaminated with hydrocarbons; SMP_C: Non-rhizospheric sediment or soil collected in area with vegetation; SMP_D: Rhizospheric Soil; SMP_E: parts of the native and associated mangrove plant species (roots/stems/leaves/fruits)

The partial and total relative abundances of fungal taxa across different sample types were obtained and are shown in Fig. 3 and **Supplementary Material **9. The dominant phylum in all samples is Ascomycota (91.05%), followed by Basidiomycota (7.23%), Basidiobolomycota (1.01%), Chytridiomycota (0.32%), Rozellomycota (0.26%), and other phyla with less than 0.1% relative abundance.

Data analysis of all samples revealed a notable predominance of unclassified readings: 95.6% at the species level, 90.03% at the genus level, 87.1% at the family level, 74.17% at the order level, and 69.88% at the class level (**Supplementary Material 9**).

Considering the results including and excluding the unclassified fungal taxa, the ten genera of fungi were the most abundant in all the samples are *Phaeophleospora* (2.38%; 23.86%), followed by *Psathyrella* (1.73%; 17.33%), *Aspergillus* (1.50%; 15.01%), *Penicillium* (0.71%; 7.11%), *Halosarpheia* (0.37%; 3.71%), *Candida* (0.34%; 3.42%), *Talaromyces* (0.15%; 1.52), *Trichoderma* (0.14%; 1.35%), *Pseudallescheria* (0.11%; 1.13%), and *Corollospora* (0.11%; 1.12%) (**Supplementary Material 9**). Considering only those classified, 74 genera were abundant, with percentages greater than 0.1%, and the rest (303 genera) were present in very low total relative abundances (< 0.1%). Excluding the unclassified, only these ten genera were abundant, with percentages greater than 0.1%.

#### Richness and relative abundance of fungi in sediments kept in microcosms without vegetation (SMP_A)

Of the 11 phyla and 377 genera classified, six phyla and 46 genera were recorded in sediments kept in microcosms without vegetation (SMP_A). The phylum *Ascomycota* (65.19%) was prominent (Fig. 3a), with genera *Penicillium* (9.31%), *Aspergillus* (9.20%), *Gliocephalotrichum* (8.70%), *Strelitziana* (6.35%), *Lasiobolidium* (5.64%), *Candida* (4.71%), *Verruculina* (4.46%) and other genera < 4%, when considering the most abundant genera excluding the unclassified ones (Fig. 3c). *Basidiobolomycota* (32.79%) was the second most abundant phylum, and *Basidiomycota* (1.08%), *Rozellomycota* (0.55%), *Chytridiomycota* (0.31%), and *Mucoromycota* (0.07%) were present in small proportions (Fig. 3c and **Supplementary Material **9). As the partial relative abundance percentage of those unclassified at the genus level was high (97.26%), the most abundant genera had percentages lower than 0.5%, so they were not represented in the bar graphs (Fig. 3b and **Supplementary Material **9).

#### Richness and relative abundance of fungi in sediments kept in microcosms without vegetation and contaminated with hydrocarbons (SMP_B)

In sediments kept in microcosms without vegetation and contaminated with hydrocarbons (SMP_B), six phyla and 31 genera were identified. Fungal communities were dominated by *Ascomycota* (87.18%) (Fig. 3a and **Supplementary Material **9), including *Penicillium* (2.61%) and *Aspergillus* (1.3%) when considering the most abundant genera, including the unclassified ones (91.5%) (Fig. 3b and **Supplementary Material **9). Thus, when the unclassified were excluded, the most abundant genera of *Ascomycota* were *Penicillium* (30.68%), *Aspergillus* (15.3%), *Saccharomycopsis* (4.4%), and *Verticillium* (4.05%). *Basidiobolomycota* (6.95%) was the second most abundant phylum, and *Basidiomycota* (2.28%), *Chytridiomycota* (1.92%), *Mucoromycota* (1.26%), and *Rozellomycota* (0.4%) were present in small proportions (Fig. 3c and **Supplementary Material **9). In *Basidiomycota*, the genera *Colacogloea* (0.53% and 6.19%) and *Rhodotorula* (0.56% and 6.6%) were abundant, either including (Fig. 3b and **Supplementary Material **9) or excluding the unclassified ones (Fig. 3c and **Supplementary Material **9). The genus *Rhizopus* (*Mucoromycota*) was also dominant in this substrate, with a small percentage (0.53%) including the unclassified ones (Fig. 3b and **Supplementary Material **9) and a notably abundant percentage (6.23%) excluding the unclassified taxa (Fig. 3c and **Supplementary Material **9).

#### Richness and relative abundance of fungi in non-rhizosphere sediment/soil collected in an area with vegetation (SMP_C)

Mycological analysis of the SMP_C sample (non-rhizosphere sediment/soil collected in a vegetated area) revealed a fungal community made up of 10 phyla and 190 genera. *Ascomycota* (85.32%) and *Basidiomycota* (13.54%) had the highest relative partial abundances of the fungal community, followed by *Chytridiomycota* (0.44%) and *Rozellomycota* (0.59%) (Fig. 3a and **Supplementary Material **9). When considering the taxa not classified at more specific taxonomic levels (92.68%), the most abundant genera were *Candida* (1.07%, *Ascomycota*), *Psathyrella* (0.97%, *Basidiomycota*), *Penicillium* (0.87%, *Ascomycota*), *Phaeophleospora* (0.57%, *Ascomycota*), and the other genera had a relative abundance of less than 0.5% (Fig. 3b and **Supplementary Material **9). Excluding the unclassified taxa, the analysis of the taxonomic composition at genus level revealed *Candida* (14.7%, *Ascomycota*), *Psathyrella* (13.29%, *Basidiomycota*), *Penicillium* (11.84%, *Ascomycota*), *Phaeophleospora* (7.8%, *Ascomycota*), and *Halorosellinia* (4.48%, *Ascomycota*) as the most abundant genera. The other genera accounted for less than 4% of the community (Fig. 3c and **Supplementary Material **9).

#### Richness and relative abundance of fungi in rhizosphere soil (SMP_D)

All 11 classified phyla and 225 genera were recorded in the rhizosphere soil (SMP_D). *Ascomycota* (92.28%) was dominant, followed by *Basidiomycota* (6.17%), *Chytridiomycota* (0.75%), and *Rozellomycota* (0.55%) (Fig. 3a and **Supplementary Material **9). When considering the taxa unclassified at more specific taxonomic levels (84.07%), the most abundant genera were: *Aspergillus* (4.65%, *Ascomycota*), *Psathyrella* (3.62%, *Basidiomycota*), *Halosarpheia* (1.27%, *Ascomycota*), and *Penicillium* (1.05%, *Ascomycota*). The other genera had a relative abundance of less than 0.5% (Fig. 3b and **Supplementary Material **9). Excluding the unclassified taxa, the analysis of taxonomic composition at the genus level showed that the same genera were dominant, but in higher percentages, such as *Aspergillus* (29.23%, *Ascomycota*), *Psathyrella* (22.76%, *Basidiomycota*), *Halosarpheia* (8%, *Ascomycota*), and *Penicillium* (6.62%, *Ascomycota*). The other genera accounted for less than 4% of the community (Fig. 3c and **Supplementary Material **9).

#### Richness and relative abundance of fungi in leaves, fruits, roots, stems, and barks of mangrove trees and shrubs (SMP_E)

In fungal communities of SMP_E samples, seven phyla and 236 genera were identified (**Supplementary Material **9). *Ascomycota* (93.34%) was dominant, followed by *Basidiomycota* (6.58%) and other phyla with a relative abundance below 0.1% (Fig. 3a and **Supplementary Material **9). Including the unclassified ones at genus level (91.78%), *Phaeophleospora* (*Ascomycota*) and *Psathyrella* (*Basidiomycota*) were the most abundant genera in this substrate (SMP_E) with a small percentage of 4.11% and 1.16%, respectively (Fig. 3b and **Supplementary Material **9). The other genera had a relative abundance of less than 0.5%. By excluding the unclassified taxa from the data analyses, *Phaeophleospora* (50%), *Psathyrella* (14.13%), *Penicillium* (5.61%), and *Aspergillus* (4.59%) were dominant, with higher percentages. The other genera accounted for less than 4% of the community (Fig. 3c and **Supplementary Material **9).

#### Shareability and uniqueness of fungal genera in distinct substrates

**Fig. 4:**
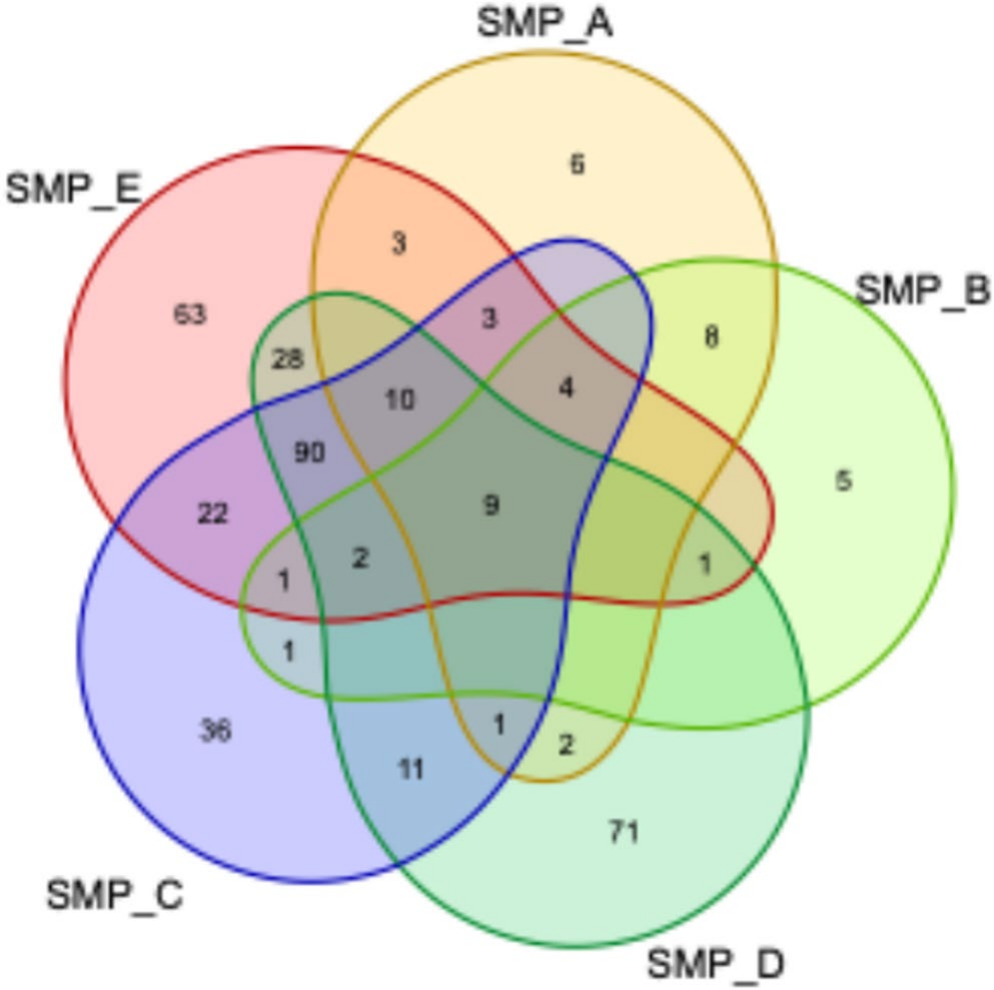
Number of shared and exclusive genera of the fungal community obtained by metagenomic analyses of ITS region amplicons. Sample type: (SMP_A: Sediments kept in microcosms without vegetation; SMP_B: Sediments kept in microcosms without vegetation and contaminated with hydrocarbons; SMP_C: Non-rhizospheric sediment or soil collected in areas with vegetation; SMP_D: Rhizospheric Soil; SMP_E: parts of the native and associated mangrove plant species (roots/stems/leaves/fruits)

In the comparative analysis, all five sample types, both the environmental compartments and the microcosms (SMP_A, SMP_B, SMP_C, SMP_D and SMP_E), shared nine fungal genera, comprising both unicellular (yeasts) (*Candida* and *Kazackstania*) and mycelial fungi (*Aspergillus*, *Cladophialophora*, *Colletotrichum*, *Ganoderma*, *Penicillium*, *Rhizopus*, and *Talaromyces*) (Figs. 3 and 4, **Supplementary Material **10).

The three environmental compartments (SMP_C, SMP_D, and SMP_E) shared most of the fungal genera (*n* = 90), but among them the shareability differed: SMP_D and SMP_E (*n* = 28), SMP_C and SMP_D (*n* = 11), SMP_C and SMP_E (*n* = 22). Furthermore, the environmental compartments displayed a higher number of unique genera, such as SMP_E (*n* = 63), SMP_C (*n* = 36), and fewer in SMP_D (*n* = 71), but even in microcosm studies, unique fungal genera have also been found.

In the two microcoms samples type (SMP_A and SMP_B), six (*Gliocephalotrichum*, *Kondoa*, *Leptosphaeria*, *Myrmecridium*, *Scleropezicula*, and *Zygosaccharomyces*) *and* 5 (*Colacogloea*, *Lactarius*,* Mycoarthris*,* Pyxidiophora*, and *Scheffersomyces)* unique fungal genera were also retrieved, respectively. (Fig. 4 and **Supplementary Material **10). But when comparing these two microcosm samples (SMP_A and SMP_B), eight genera are shared between them (*Ilyonectria*, *Lasiobolidium*, *Lichtheimia*, *Mucor*, *Rhizomucor*, *Rhodotorula*, *Saccharomycopsis*, and *Verticillium*) (Fig. 4 and **Supplementary Material **10).

The sharing between environmental compartments and microcosms (SMP_C and SMP_B (*n* = 1: *Phanerochaete)*; SMP_D and SMP_A (*n* = 2: *Pseudopithomyces* and *Ramularia)*; SMP_E and SMP_A (*n* = 3: *Peziza*, *Sporobolomyces*, and *Strelitziana*) was lower than the sharing of only environmental compartments or only microcosms and can be seen in Fig. 4 and **Supplementary Material **10.

### Alpha diversity

**Fig. 5:**
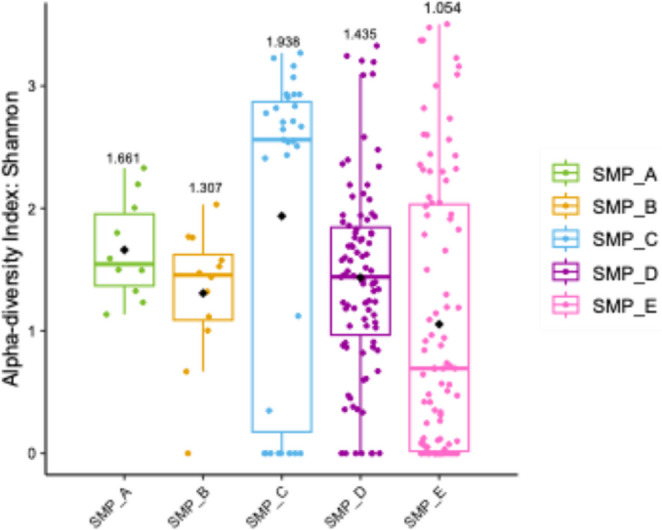
Alpha diversity of samples from mangroves at the taxonomic genus level. Sample type: (SMP_A: Sediments kept in microcosms without vegetation; SMP_B: Sediments kept in microcosms without vegetation and contaminated with hydrocarbons; SMP_C: Non-rhizospheric sediment or soil collected in areas with vegetation; SMP_D: Rhizospheric Soil; SMP_E: parts of the native and associated mangrove plant species (roots/stems/leaves/fruits)

Alpha-diversity analysis revealed variable levels of diversity among the sample groups. The SMP_E group presented the lowest mean Shannon index (1.054), indicating reduced microbial diversity in this condition. Similarly, the SMP_B, SMP_D, and SMP_A groups showed relatively low diversity, with mean Shannon indices of 1.307, 1.435, and 1.661, respectively. In contrast, the SMP_C group exhibited higher alpha diversity, with a mean Shannon index of 1.938, suggesting a more complex microbial community in these environments (Fig. 5).

Pairwise ADONIS (PERMANOVA) tests revealed statistically significant differences in the community of fungal genera between various groups of samples (SMP). Specifically, the microbial communities in the following comparisons differed significantly after adjustment: **SMP_B** vs. **SMP_C** (p.adjusted = 0.002), **SMP_A** vs. **SMP_C** (p.adjusted = 0.011), between the samples **SMP_E** and both **SMP_C and SMP_D** (p.adjusted = 0.001 for both), and **SMP_C** vs. **SMP_D** (p.adjusted = 0.001) (Table 7). These comparisons indicate that microbial communities differed meaningfully between these groups. However, other pairwise comparisons, for example, between sample **SMP_B** and both **SMP_A**, **SMP_E**, and SMP**_D**, **and** between sample **SMP_A** and both **SMP_E** and **SMP_D**, did not show significant differences, aligning with the closer Shannon index values observed in Fig. 5; Table 7.

**Table 7:** Permanova analysis of alpha diversity. ns: No significant difference; * : p.adjusted < 0.05; **: p.adjusted < 0.01. Sample type: (SMP_A: Sediments kept in microcosms without vegetation; SMP_B: Sediments kept in microcosms without vegetation and contaminated with hydrocarbons; SMP_C: Non-rhizospheric sediment or soil collected in areas with vegetation; SMP_D: Rhizospheric Soil; SMP_E: parts of the native and associated mangrove plant species (roots/stems/leaves/fruits)

Pairs	Df	SumsOfSqs	F.Model	R2	*p*.value	p.adjusted	sig	p.permanova
SMP_B vs. SMP_A	1	0.02869128255	1.546937278	0.07528797391	0.2174	1	ns	0.000999000999
SMP_B vs. SMP_E	1	0.396835836	2.703506288	0.0326891375	0.0698	0.698	ns	0.000999000999
SMP_B vs. SMP_C	1	0.5886682646	0.5886682646	0.3731878343	2.00E-04	0.002	**	0.000999000999
SMP_B vs. SMP_D	1	0.01971982509	0.4046333514	0.00433204796	0.6308	1	ns	0.000999000999
SMP_A vs. SMP_E	1	0.4655128045	3.157271577	0.03842960601	0.0431	0.431	ns	0.000999000999
SMP_A vs. SMP_C	1	0.3340032692	11.58482199	0.2720411041	0,0011	0.011	*	0,000999000999
SMP_A vs. SMP_D	1	0.05023390214	1.041495544	0.01119388224	0.3141	1	ns	0.000999000999
SMP_E vs. SMP_C	1	1.677605957	12.56406806	0.1201566494	1.00E-04	0.001	**	0.000999000999
SMP_E vs. SMP_D	1	1.120659613	10.83286883	0.0661214621	1.00E-04	0.001	**	0.000999000999
SMP_C vs. SMP_D	1	1.148877923	23.77584827	0.1846297158	1.00E-04	0.001	**	0,000999000999

### Beta diversity

**Fig. 6:**
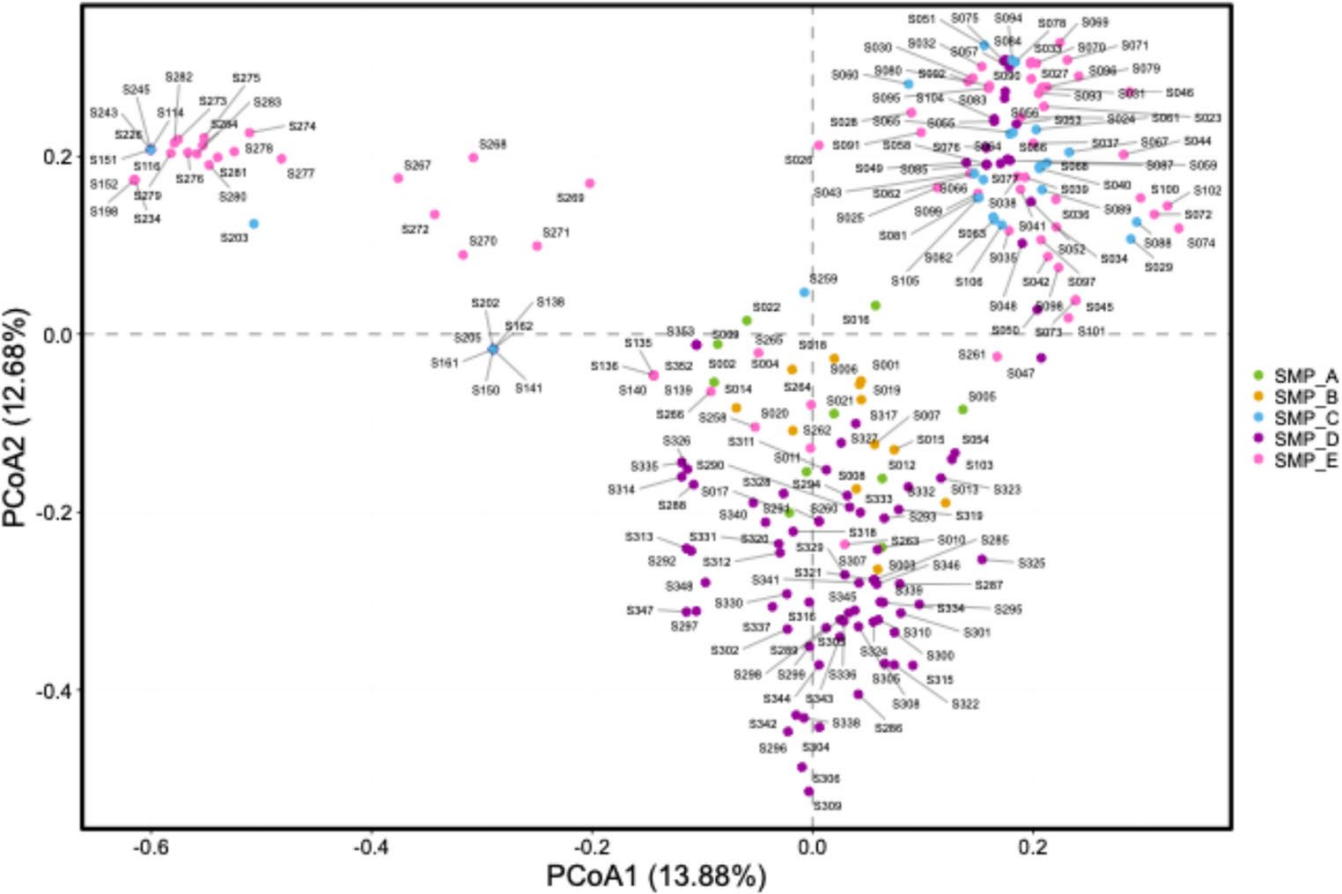
Beta diversity of samples from mangroves at the taxonomic genus level. Sample type: (SMP_A: Sediments kept in microcosms without vegetation; SMP_B: Sediments kept in microcosms without vegetation and contaminated with hydrocarbons; SMP_C: Non-rhizospheric sediment or soil collected in areas with vegetation; SMP_D: Rhizospheric Soil; SMP_E: parts of the native and associated mangrove plant species (roots/stems/leaves/fruits)

Beta diversity, represented by a Principal Coordinates Analysis (PCoA), was used to visualize dissimilarity in microbial community composition among the different groups of samples (SMP) based on ITS genus-level data. The two principal coordinates, PCoA1 and PCoA2, explained 13.88% and 12.68% of the total variation, respectively. The Fig. 6 clearly illustrates the different grouping patterns that were revealed by the ordination: The SMP_A and SMP_B samples (represented by the colors green and orange, respectively) were grouped close to the origin in the lower left and right quadrants (both samples) and some individual samples in the central portions (only SMP_A) and these two samples showed partial overlap, indicating some degree of similarity in community composition. In contrast, the SMP_C, SMP_D, and SMP_E samples (represented by light blue, purple, and pink, respectively) showed greater overlap, predominantly occupying the central, upper, and lower-right quadrants, suggesting greater similarity in community composition. Although groups of samples in the upper left quadrant grouped individual samples from the SMP_C (light blue color) and SMP_E (pink color) samples, individual samples overlap from the SMP_D (purple color) and SMP_E (pink color) samples in the lower left quadrants.

**Fig. 7:**
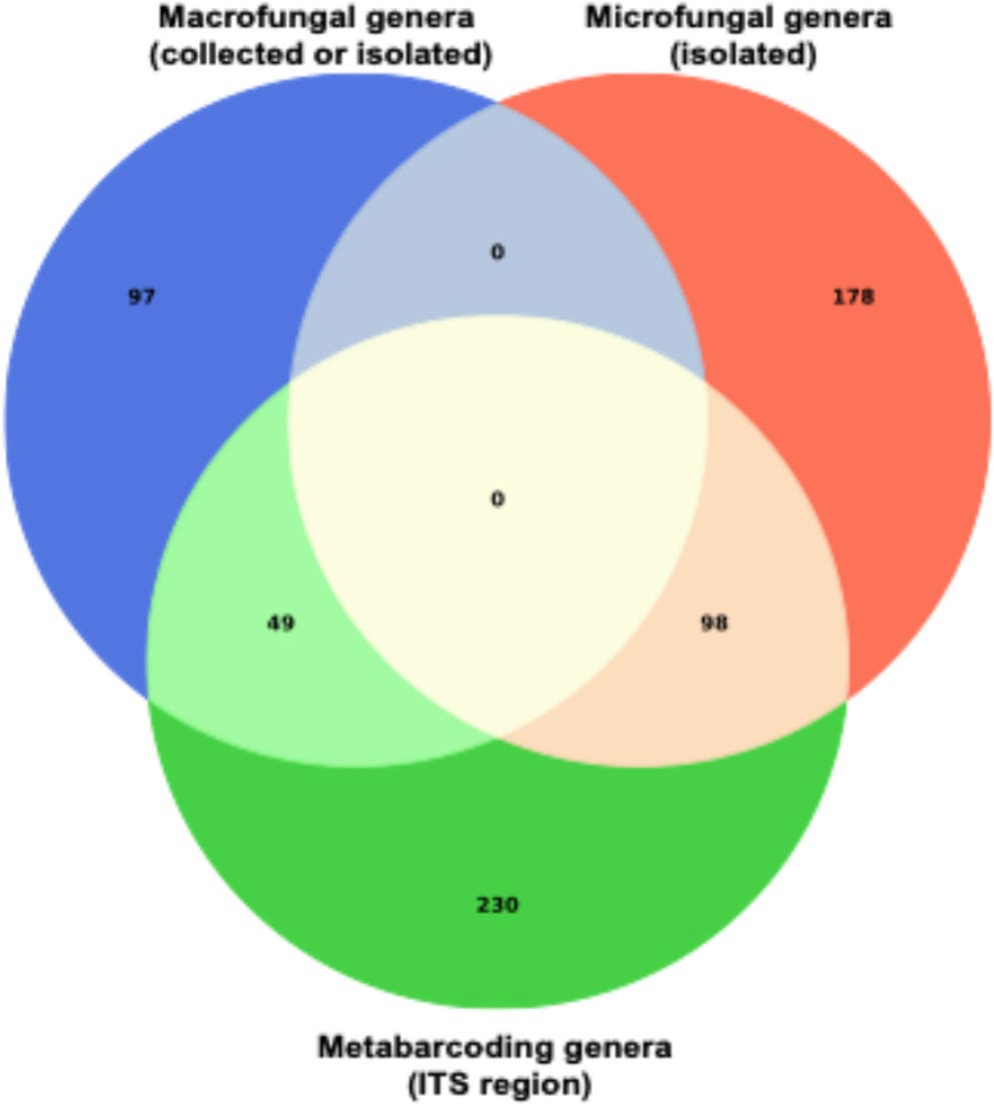
Shareability and uniqueness patterns of macrofungi from culture-derived isolates and field-collected specimens, microfungi culture-derived isolates, and metabarcoding (ITS region)

The differences in microbial community composition at the genus level shown in Fig. 7 were confirmed by PERMANOVA, which indicated statistically significant differences in most pairwise comparisons (Table 8). The SMP_B samples showed significant differences compared with SMP_E (p-adjusted = 0.007), SMP_C (p-adjusted = 0.002), and SMP_D (p-adjusted = 0.002). Similarly, SMP_A differed significantly from SMP_E (p. adjusted = 0.018), SMP_C (p. adjusted = 0.048), and SMP_D (p. adjusted = 0.023). Differences were also observed between the environmental samples SMP_E vs. SMP_C, SMP_E vs. SMP_D, and SMP_C vs. SMP_D (p-adjusted = 0.001 for all comparisons), further supporting the influence of the environmental site or plant compartment on the microbial community structure (Table 8).

**Table 8:** Permanova analysis of beta diversity. ns: No significant difference; * : p.adjusted < 0.05; **: p.adjusted < 0.01. Sample type: (SMP_A: Sediments kept in microcosms without vegetation; SMP_B: Sediments kept in microcosms without vegetation and contaminated with hydrocarbons; SMP_C: Non-rhizospheric sediment or soil collected in areas with vegetation; SMP_D: Rhizospheric Soil; SMP_E: parts of the native and associated mangrove plant species (roots/stems/leaves/fruits)

Pairs	Df	SumsOfSqs	F.Model	R2	*p*.value	p.adjusted	sig	p.permanova
SMP_B vs. SMP_A	1	0.4600589586	1.072491696	0.05089534317	0.332	1	ns	0.000999000999
SMP_B vs. SMP_E	1	1.312964326	2.965074948	0.03057879292	7.00E-04	0.007	**	0.000999000999
SMP_B vs. SMP_C	1	1.194460363	3.039844025	0.07592047618	2.00E-04	0.002	**	0.000999000999
SMP_B vs. SMP_D	1	1.090149261	2.547216466	0.02558802302	2.00E-04	0.002	**	0.000999000999
SMP_A vs. SMP_E	1	1.127652337	2.533185408	0.02679678461	0.0018	0.018	*	0.000999000999
SMP_A vs. SMP_C	1	0.9215805262	2.325807535	0.06231097701	0.0048	0.048	*	0.000999000999
SMP_A vs. SMP_D	1	0.8357833551	1.943987216	0.02005268477	0.0023	0.023	*	0.000999000999
SMP_E vs. SMP_C	1	1.36239087	3.165426022	0.02822104934	1.00E-04	0.001	**	0.000999000999
SMP_E vs. SMP_D	1	3.424282985	7.832127263	0.04429131394	1.00E-04	0.001	**	0.000999000999
SMP_C vs. SMP_D	1	2.039877442	4.881453159	0.04176413817	1.00E-04	0.001	**	0.000999000999

### Shareability and uniqueness patterns of collected or isolated from culture macrofungi, isolated from culture microfungi, and metabarcoding

As shown in the Venn diagram (Fig. 7), metabarcoding (ITS region) exclusively identified the highest number of fungal genera (*n* = 230), whereas pure culture isolation yielded 178 fungal genera unique to microfungi and 97 unique to macrofungi (**Supplementary Material **7). Shared fungal genera were observed only between amplicon sequencing (ITS region) and culture-based isolates. Specifically, 49 fungal genera were shared between amplicon sequencing and macrofungi (including both culture-derived isolates and field-collected specimens), and 98 fungal genera were shared between amplicon sequencing (ITS region) and microfungal isolates (**Supplementary Material **7).

### Ecological traits of fungal taxa

The functional characterization of fungal taxa identified by sequencing the ITS region revealed a wide diversity of ecological strategies. From 377 genera identified and 763 OTUs obtained in the original dataset (**Supplementary Material **9), 363 fungal genera and 549 OTUs displayed functional information available in the FungalTraits database (**Supplementary Material **11).

**Fig. 8:**
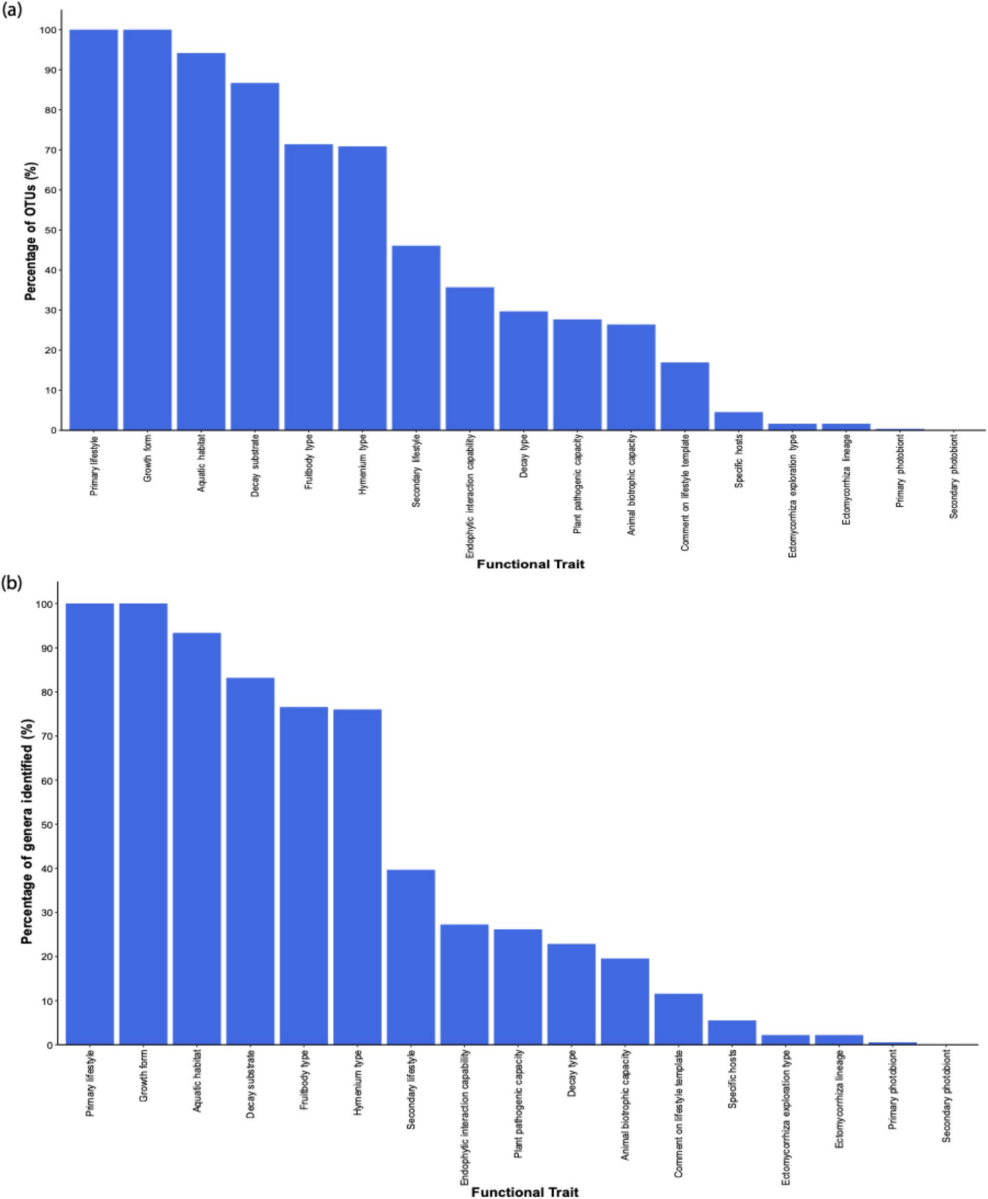
Functional traits of fungi occurring in mangroves worldwide. (**a**) Percentage of OTUs (%) for each fungal trait; (**b**) Percentage of Genera Identified (%) for each fungal trait

Seventeen distinct functional characteristics were identified, encompassing ecological, morphological, and physiological aspects of each genus, as inferred from the ITS region. These characteristics included: primary (**1**) and secondary (**2**) lifestyle, additional notes on lifestyle (**3**), endophytic interaction capacity (**4**), pathogenic potential in plants (**5**), decay substrate (**6**), decay type (**7**), occurrence in aquatic habitats (**8**), animal biotrophic capacity (**9**), host specificity (**10**), growth form (**11**), sporome (fruiting body type) (**12**), hymenium type (**13**), mode (**14**) and lineage (**15**) of ectomycorrhizal exploitation, and the presence of primary (**16**) and secondary (**17**) photobionts. Among the functional characteristics evaluated, the most widely represented were primary lifestyle and growth form, which were attributed to 100% of the OTUs analyzed (*n* = 549) and 100% of the classified genera (*n* = 363), followed by aquatic habitat (517 OTUs, 94.17%; 339 classified genera, 93.39%) and decay substrate (476 OTUs, 86.70%; 302 classified genera, 83.2%). The fruiting body type (392 OTUs, 71.40%; 278 classified genera, 76.58%) and hymenium type (389 OTUs, 70.86%; 276 classified genera, 76.03%) traits were also highly frequent (Fig. 8a and b). Less frequently, the remaining 11 functional traits were identified and are presented in Fig. 8.

#### Primary lifestyle

**Fig. 9:**
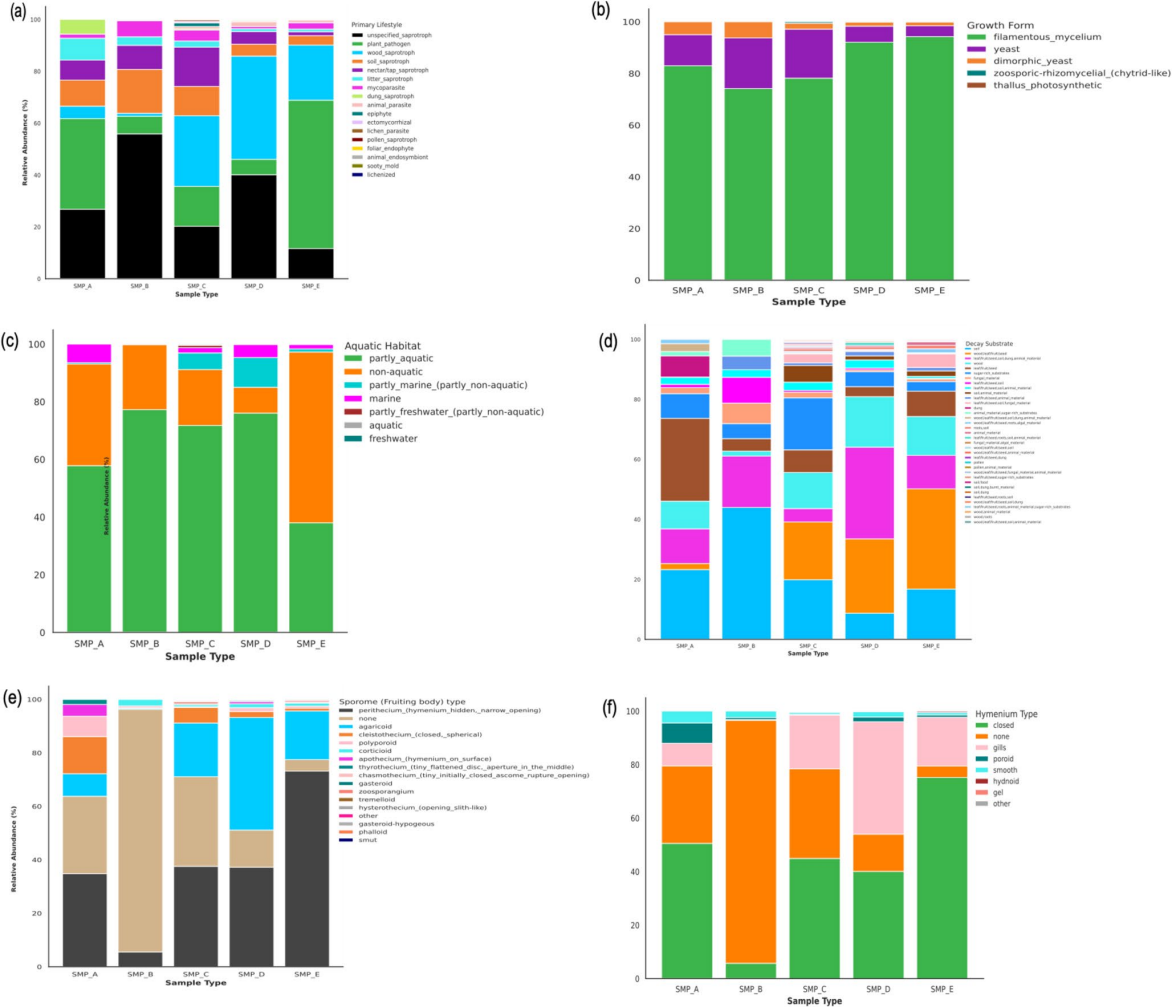
The relative abundance of fungal genus functions in terms of primary lifestyle and growth form from the FungalTraits database of sample types of mangroves. (**a**) Primary lifestyle; (**b**) Growth form; (**c**) Aquatic habitat; (**d**) Decay substrate; (**e**) Fruiting body (Sporome) type; (**f**) Hymenium type. Sample type: (SMP_A: Sediments kept in microcosms without vegetation; SMP_B: Sediments kept in microcosms without vegetation and contaminated with hydrocarbons; SMP_C: Non-rhizospheric sediment or soil collected in areas with vegetation; SMP_D: Rhizospheric Soil; SMP_E: parts of the native and associated mangrove plant species (roots/stems/leaves/fruits)

Analysis of the relative abundance of primary fungal lifestyles revealed markedly variable community composition across sample types, with consistent dominance by plant pathogens and saprotrophs. The most notable pattern emerged in the **SMP_E** sample, where plant pathogens constituted the majority, accounting for an impressive 57.21% of relative abundance, and in **SMP_A**, where this group was the most abundant (34.96%). In contrast, samples **SMP_B** and **SMP_D** were dominated by decomposer organisms: **SMP_B** was led by unspecified saprotrophs (55.88%), while **SMP_D** showed a combined abundance of unspecified saprotrophs (40.11%) and wood saprotrophs (39.83%), indicating a strong influence of woody substrate decomposition in this niche. In the **SMP_C** samples, a more balanced fungal community was found, but wood saprotrophs (27.22%) and unspecified saprotrophs (20.25%) were the most prominent traits. Mutualistic lifestyles, such as ectomycorrhizal and lichenized, were consistently underrepresented across all samples (each below 1%), highlighting the predominance of saprotrophic and pathogenic trophic strategies in the studied environment (Fig. 9a and Supplementary Material 11).

#### Growth form

Functional analysis of the fungal community, based on growth forms, revealed that filamentous fungi were dominant across sampled niches, consistently representing the most abundant morphological trait. This form of growth was present at high abundance across all five samples, with **SMP_E** (94.28%), **SMP_D** (92.10%), **SMP_A** (83.01%), **SMP_C** (78.2%), and **SMP_B** (74.16%). Therefore, this analysis suggests that mycelial growth, which is essential for penetrating and degrading complex substrates (such as lignin and cellulose), is the predominant ecological strategy in these environments. The combined abundance of unicellular forms (yeasts) varied significantly among samples, with the highest values in **SMP_B** (25.84%) and **SMP_A** (16.98%). When these traits were separated, **SMP_B** showed that typical yeast accounted for 19.61%, the second most abundant group. Similarly, in **SMP_A**, typical yeast represented 12.02% of the community. In both cases, dimorphic yeast, which is often associated with parasitic niches or environmental stress conditions, had smaller contributions, with 6.23% in **SMP_B** and 4.96% in **SMP_A**. The low representation of highly specialized growth forms, such as zoosporic-rhizomycelial (typically associated with aquatic environments or the decomposition of recalcitrant materials in specific niches) and photosynthetic thallus (characteristic of pioneer or oligotrophic communities, such as lichens), displayed relative abundances below 1% in all samples. This absence of specialized trophic and dispersal niches reinforces the conclusion that the growth form of the fungal community in this ecosystem is predominantly driven by mycelial growth (Fig. 9b and Supplementary Material 11).

#### Aquatic habitat

Analysis of fungal community traits based on aquatic habitats revealed distinct patterns of adaptation to the humidity and salinity of the mangrove ecosystem. The **SMP_A**, **SMP_B**, **SMP_C**, and **SMP_D** samples were consistently dominated by fungi classified as “partly_aquatic,” with relative abundances of 57.82% (**SMP_A**), 77.28% (**SMP_B**), 71.77% (**SMP_C**), and 76.08% (**SMP_D**). A distinct dominance pattern emerged in the **SMP_E** sample, which represents explicitly plant parts (plant substrate), in stark contrast to the other samples. In this niche, the “non-aquatic” category was overwhelmingly dominant, accounting for 59.20% of the community, whereas partially aquatic fungi accounted for 38.06%. This reversal of dominance not only suggests but also confirms that the fungal community in **SMP_E** is typically aeroterrestrial, composed predominantly of taxa associated with leaf surfaces or stems (e.g., endophytes and epiphytes). The marine influence on fungal composition is identified by the concentration of taxa with salt tolerance or dependence, particularly in the **SMP_D** (rhizosphere soil) and **SMP_A** (sediments in microcosms without vegetation) samples. Although the relative abundance (%) of these fungi is small, their presence confirms the strength of salt selectivity and the niche structure of the mangrove. The **SMP_A** sample, composed of sediments kept in microcosms, had the highest proportion of purely marine fungi (6.42%). This result is particularly noteworthy, as it indicates that even under controlled experimental conditions isolated from the tide, the saline legacy and the fungal community highly adapted to the original marine environment were maintained in the substrate. On the other hand, **SMP_D** (rhizospheric soil), a zone of intense water exchange, exhibited the highest combined marine influence (14.84% marine and partially marine taxa), which is consistent with adaptation to a fluctuating saline environment (Fig. 9c and Supplementary Material 11).

#### Decay substrate

Analysis of fungal traits based on decay substrates revealed trophic strategies that directly reflect the environmental pressures in each niche. The **SMP_B** sample (sediments maintained in microcosms without vegetation and contaminated with hydrocarbons) showed the highest dominance of the ‘soil’ category (43.92%), indicating that the fungi are essentially generalist soil saprotrophs. This high abundance is ecologically significant, as it suggests a robust decomposer community capable of processing recalcitrant organic matter in the sediment, including contaminant hydrocarbons, a sign of bioremediation potential. In contrast, **SMP_C** (non-rhizospheric sediment or soil collected in vegetated areas) exhibited a more balanced dominance of the ‘soil’ substrate (19.92%), with lower abundances in categories such as ‘wood, leaf/fruit/seed’ (19.27%) and ‘sugar-rich substrates’ (17.37%). Notably, the **SMP_D** sample (rhizospheric soil) showed the greatest preference for highly mixed and nutrient-rich categories, with ‘leaf/fruit/seed, soil, dung, animal_material’ reaching 30.51%, suggesting that rhizospheric soil acts as a degradation hotspot, concentrating the runoff of various organic nutrients, in addition to the presence of microorganisms that degrade organic matter. dung, animal_material reaching 30.51%, suggesting that rhizospheric soil acts as a degradation hotspot, concentrating the flow of various organic nutrients, in addition to the presence of wood-decaying fungi (24.82% in combination). In **SMP_A** samples (sediments kept in microcosms without vegetation), the highest abundance was observed in the ‘leaf/fruit/seed’ category (27.65%), closely followed by ‘soil’ (23.29%). This distribution suggests that the remaining fungal community specializes in degrading the most readily accessible organic matter (e.g., leaf litter and fruit) present in the original sediment, even in the absence of living plants in the microcosm. Finally, the **SMP_E** samples (part of the plant) also confirmed the importance of woody and organic decomposition, with SMP_E dominated by combinations of ‘wood’ (total of 46.34%), with 33.36% for the wood, leaf/fruit/seed category, and 12.97% for the wood category (Fig. 9d and Supplementary Material 11).

#### Sporome (Fruiting body) type

Analysis of sporome (fruiting body) types revealed a strong dichotomy in the fungal community, reflecting crucial differences in reproduction and dispersal among mangrove microenvironments. Most samples were dominated by two main types: perithecia and the category “none” (no defined fruiting body). The **SMP_E** sample showed the highest specialization, with perithecia dominating at 73.20% relative abundance. This result, combined with the knowledge that **SMP_E** represents plant parts (plant substrate), suggests that the fungal community in this niche is primarily composed of lignicolous/endophytic ascomycetes that produce perithecia for ascospore release, a mechanism highly adapted to dispersal in humid, structured environments. In contrast, **SMP_B** (sediments kept in microcosms without vegetation and contaminated with hydrocarbons) was dominated by the category “none” (90.77%), indicating that the vast majority of fungi present are taxa that do not produce macroscopic fruiting bodies or that are reproductively inactive under the stress of contamination and confinement in microcosms (e.g., yeasts, anamorphic fungi, and zygomycetes). In contrast to the extremes observed (**SMP_E** and **SMP_B**), samples **SMP_A** (sediments in microcosms), **SMP_C** (non-rhizospheric soil), and **SMP_D** (rhizospheric soil) exhibited greater diversity in reproductive strategy. In **SMP_A**, the distribution was divided between the most abundant fruiting body, the perithecium (34.80%), and fungi that do not produce defined fruiting bodies (“none,” 28.97%), with agaricoids contributing 8.44%. In SMP_C samples, a similar distribution pattern was observed, but with a higher contribution from agaricoids (20.12%), while perithecia (37.58%) and the “none” category (33.48%) were also highly represented. Finally, **SMP_D** was the only sample in which the agaricoid type (42.10%) was the most abundant, exceeding perithecia (37.24%). The “none” category was less prominent in this rhizospheric soil (13.91%). This reversal in dominance (agaricoid over perithecium) in **SMP_D**, and the contribution of 20.12% in **SMP_C**, suggests that soil influenced by roots (**SMP_D**) and non-rhizospheric soil in a vegetated area **(SMP_C**) support communities of basidiomycetes that form more complex fruiting bodies and are crucial for nutrient cycling in this microenvironment (Fig. 9e and Supplementary Material 11).

#### Hymenium type

In total, eight (8) categories of functional morphological traits of the hymenium (closed, none, gills, poroid, smooth, gel, hydnoid, and other) were obtained from the FungalTraits database to characterize the functionality of fungal genera in the five different types of mangrove samples (SMP_A to SMP_E).

The relative abundance (%) revealed a high degree of functional divergence in the type of hymenium among the fungal genera of the different sample types. The results indicated relative abundance percentages greater than 40% for traits associated with spore protection and internal development (closed hymenium) in four of the five samples analyzed (**SMP_A**: 50.56%; **SMP_C**: 44.93%; **SMP_D**: 40.08%; **SMP_E**: 75.19%). In contrast, sediments kept in microcosms without vegetation and contaminated with hydrocarbons (sample **SMP_B**) were almost entirely dominated by fungi that do not form hyphae (type none, 90.77%), reflecting a community with a strong presence of non-basidiomycete taxa or microfungi. In turn, **SMP_D** differed due to the predominance of fungi with the “gills” trait (42.03%), indicating that, in this habitat, aerodynamic dispersal strategies associated with lamellae are ecologically favored (Fig. 9f and Supplementary Material 11).

## Discussion

We synthesised the worldwide ITS metabarcoding literature on mangrove fungi using a PRISMA-guided protocol and a unified bioinformatic pipeline applied to all retrievable raw reads. This combined approach allowed us to identify general patterns of taxonomic composition, community structure and functional traits across substrates, geographies and experimental settings, which we discuss below.

### Core fungal mycobiomes in mangroves

After analyzing both non-rhizospheric and rhizospheric soil in natural and microcosm conditions, as well as in distinct plant compartments (roots, stems, leaves, and fruits) all over the coastal areas in all the continents where mangroves occur, there seems to be a consistent core fungal mycobiome. It is strongly composed of *Ascomycota* taxa, which are much more relatively abundant than all the other fungal phyla. A notable feature is that ascomycota reads typically account for > 90% across all samples, a pattern consistently observed in studies worldwide (Muwawa et al. 2024). Furthermore, besides *Basidiomycota*, which is (expectedly) the second most abundant fungal phylum in mangroves, *Rozellomycota* and *Chytridiomycota* are also prominent in this environment. This is a non-trivial finding, corroborated by recent studies, and it opens new opportunities to study and better understand the ecological roles of basal fungal lineages in mangroves (Zhang et al. 2021).

Similarly to what was found for the much more inclusive taxonomic category of phylum, there also seems to be a core fungal microbiome in mangroves at the genus level in our review. A total of 10 highly abundant fungal genera recurrently occur in the samples all over the world. Besides the almost ubiquitous and highly ecologically successful *Ascomycota* genera such as *Aspergillus*, *Penicillium*, *Talaromyces*, *Candida*, and *Trichoderma*, other genera, including *Phaeophleosopora*, *Pseudallescheria*, and *Corollospora*, as well as the Basidiomycota genus *Psathyrella*, comprise a significant portion of the total relative read abundance. *Candida* has been reported as the dominant fungal genus in mangrove soils, waters, and plants (and even invertebrates) worldwide (Nimsi et al. 2023). *Aspergillus*, *Penicillium*, and *Talaromyces* are also dominant in mangrove sediments, as well as *Trichoderma*, but at a lesser level (Britto Martins de Oliveira et al., 2025). *Corollospora* is also frequently reported as a common fungal genus in mangroves and is among the most speciose in this environment (Devadatha et al. 2021). *Pseudallescheria* and *Psathyrella* have been isolated from mangrove sediments using culturomics methods, such as the fungal enrichment culture method (FECM) and fungal isolation chips (FiChips) (Li et al. 2023). *Psathyrella* has also been recorded as a collected macrofungi in mangrove soil and vegetation (Baltazar et al. 2009; Ghate and Sridhar 2016). Furthermore, *Phaeophleospora* has recently been identified as a keystone taxon in maintaining microbial network topology through cross-domain interactions in subsurface mangrove sediments (Ng et al. 2025).

Although in our study, both ascomycota *Halorosellinia* and *Halosarpheia* are among the dominant genera in non-rhizospheric and rhizospheric sediment, respectively, they are also commonly associated, by both direct field collection and/or traditional isolation, with mangrove vegetation, especially on stems and aerial roots (Devadatha et al. 2021; Palit et al. 2022).

### Correspondence between metabarcoding knowledge and direct field collection and traditional isolation methods

Although the first studies of fungi occurring in mangroves were from the first half of last century, in Puerto Rico (Stevens 1920) and Australia (Cribb and Cribb 1955), almost all the studies were mainly published since the last decades of the past century and the first years of the current century (Kohlmeyer 1969; Hyde et al. 1988; Schmit and Shearer 2004). A synthesis of the studies comprising global fungal diversity in mangroves by (i) both active field collections and (ii) traditional culturing of collected mangrove substrates under standardized lab conditions has been quite recently published (Devadatha et al. 2021); however, it does not include ITS metabarcoding studies, and therefore, our study fills this gap.

Those studies were mainly focused on microfungi and reported macrofungi only very occasionally. Hence, we also conducted another global-level literature review (outside the scope of our primary systematic review) to identify all possible studies reporting macrofungi in mangroves (Supplementary Material 11). The central idea is to relate the ITS metabarcoding findings to current knowledge of fungal diversity in mangroves worldwide, derived from direct macrosporome field collections, as well as to microfungi widely reported in the aforementioned studies. Approximately 33–35% of the genera retrieved in ITS metabarcoding studies also occur in studies specifically dedicated to both macrofungi (Baltazar et al. 2009; Ghate and Sridhar 2016) or microfungi around the world (Devadatha et al. 2021), which clearly indicates that not only metabarcoding complement studies based on direct field collection and isolation from environmental substrates but also reveal many not-yet-cultured and possibly unknown taxa (Fonseca et al. 2022).

### Functional traits of fungi occurring in mangroves

Functional traits comprise phenotypic features (morphological, physiological, and behavioral - for animals) at the individual scale that respond to or interact with the environment along a continuum of ecological responses and effects (Weiss and Ray 2019). Functional-trait approaches are widely regarded as effective for evaluating the mechanisms behind community assembly patterns and ecosystem dynamics (Dawson et al. 2019). Although knowledge of fungal functional traits remains incomplete and challenging, significant efforts have been made in recent years to facilitate functional assignments and the ecological interpretation of environmental studies (Põlme et al. 2020).

As functional traits of fungi are usually conserved at the genus level and, sometimes, even at higher taxonomic levels (Zanne et al. 2020), we could retrieve relevant information using this approach to better understand ecological patterns of fungi occurring in mangroves, at least based on four functional traits that were confidently assigned to more than 80% of total fungal genera. The principal ecological role of fungi in both terrestrial (Vaz et al. 2017) and aquatic (Pagani et al. 2023) environments is the decomposition of organic matter, and this is also true for mangroves. Most of the fungi occurring in mangrove ecosystems are saprothrophs associated with the decomposition of plant necromass, which encompasses both non-woody (mainly leaves, but also flowers and fruits) and woody residues, (twigs, stems, branches, trunks and roots) (Moitinho et al. 2022) and all sort of organic matter detritus in sediments (Zuo et al. 2022), corroborating our worldwide findings on fungal traits based on ITS metabarcoding for both the primary lifestyle (all subcategories of saprotroph) and decay substrate (all subcategories of soil, woody and non-woody substrates), not only based on distinct types of environmental samples but also in studies using both unpolluted and hydrocarbonate-polluted microcosms.

Nevertheless, a quite surprising finding was a slight majority of the plant pathogen’s primary lifestyle in the SMP_E group, which unites all the samples across studies of distinct parts of the native and associated mangrove plant species. There are a few studies specifically investigating plant pathogenic fungi in mangrove plants (Rafael and Calumpong 2019; Sahibu et al. 2020; Goudarzi and Moslehi 2020). Although the USDA fungus-host database can be used as a proxy to estimate plant-associated fungi in specific mangrove plants, a considerable amount of the fungi may be endophytes, and; therefore, it is not possible to confidently associate them with plant pathogenic *primary lifestyle* (Mishra et al. 2021). Hence, there should be a probable underestimate of plant pathogenic fungi (Gilbert et al. 2002), and; thus, mangrove fungi community may constitute a plant pathogen reservoir, potentially harboring still unknown specific diseases of mangrove plants.

Associations between plant roots and symbiotrophic mycorrhizal fungi benefit most plants by improving nutrient uptake and increasing tolerance to stress (Tedersoo et al. 2020). Mangrove sediments are harsh, highly stressful environments: poorly aerated, saline, mobile, and tidally inundated, and constantly subjected to large fluctuations in most physicochemical parameters (Keith et al. 2020). Furthermore, mangrove sediments are usually low in nutrients, especially nitrogen and/or phosphorus (Lovelock et al. 2006), a condition that extensively favours the association of mycorrhizal fungi with the roots of their plant hosts (Elumeeva et al. 2018). In our world inventory based on ITS metabarcoding of fungi occurring in mangroves, the proportion of symbiotrophic mycorrhizal fungi was very low, and a quite surprising finding was the small but still significant proportion of ectomycorrhizal fungi in the SMP_C group, which consists of non-rhizospheric sediment or soil collected in areas with vegetation. Studies on ectomycorrhizal fungi in mangrove plant species are extremely scarce, but among the few conducted, at least some species were identified as ectomycorrhizal (Kundu et al. 2025). The exceedingly low proportion of endomycorrhizal fungi, even in the SMP_D group (rhizospheric sediment or soil collected in areas with vegetation), could be, at first, hard to explain because there are many studies revealing the high endomycorrhization degree of mangrove-specific plants and the identification of their associated endomycorrhizal fungi (Wang et al. 2010; Ramírez-Viga et al. 2020; Pachu and Mohan 2023). Nonetheless, this is a specific methodological issue directly related to ITS metabarcoding since the overwhelming majority of the studies are conducted using short-read sequencing platforms. The severe underestimate of *Glomeromycota* (a fungal phylum exclusively composed of endomycorrhizal fungi) in the aforementioned technique and sequencing platform is mainly due to the high variability of ITS copies within their multinucleate hyphae (Tedersoo et al. 2022).

As mangroves are in transitional terrestrial-freshwater-marine environments and comprise brackish tidal systems (Keith et al. 2020), highly relevant ecological information is the *aquatic habitat* functional traits of the fungi occurring in mangroves since it reveals not only if the fungi are non-aquatic, partly aquatic, or aquatic, but also their probable origin (marine, freshwater) and; therefore, their haloresistance and halotolerance in water (Põlme et al. 2020). Regardless of the analyzed ITS metabarcoding sample type groups, which were from direct environmental studies or microcosms studies (including from non-polluted and hydrocarbon-polluted sediments), most of the fungi were considered *partly aquatic* and, therefore, comprising residents, periodic immigrants or versatile immigrants, in accordance with their degree of adaptation, activity, and dependence on aquatic habitats (Pagani et al. 2023). Partly marine and true marine fungi were also found, especially in rhizospheric and non-rhizospheric sediments, including those from non-polluted microcosms, reinforcing their ecological relevance in mangroves (Calabon et al. 2023). Nonetheless, *non-aquatic* fungi are significantly found in ITS metabarcoding studies worldwide, encompassing the majority subcategory of *aquatic habitat* functional trait in the sample type group of plant organs (especially the aerial ones). Their high representativeness can be directly linked with the high diversity of both endophytes and epiphytes in mangrove plants (Yao et al. 2019; Zhu et al. 2023).

Growth form is related to both the basic body plan and the specific architecture of fungi, as well as their ability to vary their morphology (Nagy et al. 2017). Hence, it is one of the most fundamental traits embodying several key properties directly (size, shape, plasticity), and influencing others indirectly (nutritional and reproductive modes, longevity, extent and manner of locomotion) (Andrews 1995). Independent of the sample type groups (directly environmental or microcosm ones), in all the worldwide ITS metabarcoding we retrieved and analyzed, the filamentous-mycelium represents the great majority of all growth forms, followed by yeast and, to a lesser extent, dimorphic-yeast growth forms. The intricate, branching hyphal networks of fungal mycelia are adapted for efficient exploration of their substrate, a foraging strategy which may have driven the emergence of multicellularity in fungi (Nagy et al. 2017). Nonetheless, yeast growth form was more abundant worldwide in sediments, whether directly collected from the environment or cultivated in non-polluted or hydrocarbon-polluted microcosms, compared to samples from rhizospheric sediments or plant organs. Although yeasts have been isolated from various microhabitats in the mangrove ecosystem, including vegetation, water, sediments, and animals, their greatest abundance was usually found in sediments and water (Nimsi et al. 2023), which is consistent with our global findings from ITS metabarcoding studies. Due to their unicellular form, morphology, and limited dispersal, yeasts are adapted to liquid settings and the absorption of simple sugars, unlike filamentous fungi, which can forage across greater distances and in drier conditions. Naturally, yeasts predominantly occur in aquatic habitats or moist, sugar-rich niches, as well as in the gut microbiota of animals (Nagy et al. 2017). As mangrove sediments are usually associated with brackish water, resulting from the mixture of river and marine waters (Saravanakumar et al. 2008), yeasts are naturally more abundant in these substrates. Surprisingly, we retrieved a much higher proportion of yeast *growth form* in hydrocarbon-polluted microcosm samples (SMP-B) worldwide than in any other sample type group. Mangrove sediments worldwide are known to be heavily contaminated with oil, and the microbiota living within them are recognized for their high activity in metabolizing and detoxifying petroleum fractions (Pothayi et al. 2024). Furthermore, a recent study on the bioprospecting of unicellular fungi reported high biosurfactant-producing potential in yeasts from mangroves, indicating that these isolates have strong emulsification capacity for hydrocarbons (Silva et al. 2024).

### Study implications and recommendations for future research

One of the main advantages of conducting fungal ITS metabarcoding studies instead of traditional field collection or isolating from distinct substrates is the fact that, using metabarcoding (even despite PCR biases and primer choices), one can easily access most of the fungal diversity of any kind of environment (Vaz et al. 2017). This is especially fundamental if we need to access the macrofungal diversity since, compared to the extensive and long-lasting studies about microfungi diversity based on typical isolation techniques, there are very few studies on the diversity of macrofungi occurring in mangroves (Ghate and Sridhar 2016), and even fewer considering a global-level investigation (Baltazar et al. 2009). One of our quite surprising findings in this ITS metabarcoding review study was the identification of the basidiomycotan genus *Psathyrella* as a likely representative of the core mycobiome across in all sample type groups worldwide (at least in the studies retrieved through the quantitative re-analysis of all retrievable raw ITS0. Moreover, we could also retrieve the proportions of fruiting body (sporome) and hymenium-type *growth forms*, revealing a much higher abundance of agaricoid sporome and gill hymenium types, suggesting a probable, underestimated ecological importance of basidiomycotan species that exhibit these growth forms (Dutta et al. 2013). Furthermore, the beginning of metabarcoding studies performed by long-read 18 S–ITS–28 S metabarcoding will probably expand the retrieval of Glomeromycota as well as the early-diverging fungal lineages.

## Conclusions

Using a systematic review methodology, our work synthesized current knowledge of Internal Transcribed Spacer (ITS) metabarcoding studies of fungi conducted worldwide in mangrove ecosystems. The main patterns that emerge from our integrated analysis comprise the following: (i) there is a Core Mangrove Mycobiome, considering all substrates but also in specific ones; (ii) substrate drives fungal community structure; (iii) basidiomycotan genus *Psathyrella* as a representative of the core mycobiome; (iv) a relatively high abundant read number of agaricoid sporome and gill hymenium functional trait type, suggesting a probable high underestimated ecological importance of Basidiomycota; (v) both ascomycota *Halorosellinia* and *Halosarpheia* are among the dominant genera in non-rhizospheric and rhizospheric sediment, respectively; (vi) a higher proportion of yeast growth form functional trait in hydrocarbon-polluted microcosm samples worldwide than in any other sample type group; (vii) fungal ecological roles are dominated by saprothrophism; (viii) metabarcoding reveals a vast hidden diversity, including many taxa of macrofungi; and (ix) methodological biases impact findings, especially in the retrieval of endomycorrhizal Glomeromycota. As a perspective, we suggest long-read 18 S-ITS-28 S rDNA metabarcoding studies to retrieve endomycorrhizal Glomeromycota fungi, followed by their isolation and testing for use as fungal consortia in mangrove restoration.

## Supplementary information

Below is the link to the electronic supplementary material.

Supplementary File 1 (DOCX 212 KB)

Supplementary File 2 (DOCX 7.45 KB)

Supplementary File 3 (XLSX 1.12 MB)

Supplementary File 4 (XLSX 43.7 KB)

Supplementary File 5 (XLSX 14.9 KB)

Supplementary File 6 (XLSX 68.7 KB)

Supplementary File 7 (XLSX 378 KB)

Supplementary File 8 (XLSX 292 KB)

Supplementary File 9 (XLSX 616 KB)

Supplementary File 10 (XLSX 19.4 KB)

Supplementary File 11 (XLSX 2.90 MB)

## Data Availability

No datasets were generated or analysed during the current study.
