# Supplementary material for: Worldwide diversity and ecology of mangrove fungi: a systematic review of ITS metabarcoding studies and a quantitative, integrative analysis of raw sequence data
Source: World J Microbiol Biotechnol. 2026 Jun 13;42(7):352. doi: 10.1007/s11274-026-05065-y (PMC13264588; doi:10.1007/s11274-026-05065-y)
Supplement: Supplementary file 2 — (DOCX 7.45 KB) [file 11274_2026_5065_MOESM2_ESM.docx]

| Database | Keywords |
| --- | --- |
| Scopus | **Keywords 1**:  TITLE-ABS-KEY(mangrove) AND TITLE-ABS-KEY(microb*) AND TITLE-ABS-KEY(metagenom* OR metabarcod* OR pyrosequencing OR "ion torrent" OR "ion proton" OR illumina OR "pacific biosciences" OR sequel OR "oxford nanopore technologies" OR minion OR amplicon OR metatranscriptom* OR proteom* OR metabolom*) |
| PubMed | **Keywords 2**:  mangrove AND microb* AND (metagenom* OR metabarcod* OR pyrosequencing OR "ion torrent" OR "ion proton" OR illumina OR "pacific biosciences" OR sequel OR "oxford nanopore technologies" OR minion OR amplicon OR metatranscriptom* OR proteom* OR metabolom*) |
| Web Science | **Keywords 2**:  mangrove AND microb* AND (metagenom* OR metabarcod* OR pyrosequencing OR "ion torrent" OR "ion proton" OR illumina OR "pacific biosciences" OR sequel OR "oxford nanopore technologies" OR minion OR amplicon OR metatranscriptom* OR proteom* OR metabolom*)  **Keywords 3**:  “mangrove” AND (metagenomics OR shotgun OR (amplicons AND (16S OR 18S OR ITS OR “internal transcribed spacer”))) |

**Table S1:** Showing the key search words used in different databases
